# DDIT4 S‐Nitrosylation Aids p38‐MAPK Signaling Complex Assembly to Promote Hepatic Reactive Oxygen Species Production

**DOI:** 10.1002/advs.202101957

**Published:** 2021-07-26

**Authors:** Zilong Li, Qianwen Zhao, Yunjie Lu, Yangxi Zhang, Luyang Li, Min Li, Xuemin Chen, Donglin Sun, Yunfei Duan, Yong Xu

**Affiliations:** ^1^ Department of Hepatobiliary and Pancreatic Surgery The First People's Hospital of Changzhou The Third Affiliated Hospital of Soochow University Changzhou 213000 China; ^2^ Key Laboratory of Targeted Intervention of Cardiovascular Disease Collaborative Innovation Center for Cardiovascular Translational Medicine Nanjing Medical University Nanjing 211166 China; ^3^ Institute of Biomedical Research Liaocheng University Liaocheng 252000 China; ^4^ State Key Laboratory of Natural Medicines Department of Pharmacology China Pharmaceutical University Nanjing China

**Keywords:** liver injury, p38 signaling, post‐translational modification, reactive oxygen species (ROS), transcriptional regulation

## Abstract

Mitogen‐activated protein kinase (MAPK) signaling plays a significant role in reactive oxygen species (ROS) production. The authors have previously shown that Brahma‐related gene 1 (BRG1), a chromatin remodeling protein, contributes to hepatic ROS accumulation in multiple animal and cellular models of liver injury. Here it is reported that DNA damage‐induced transcript 4 (DDIT4) is identified as a direct transcriptional target for BRG1. DDIT4 overexpression overcomes BRG1 deficiency to restore ROS production whereas DDIT4 knockdown phenocopies BRG1 deficiency in suppressing ROS production in vitro and in vivo. Mechanistically, DDIT4 coordinates the assembly of the p38‐MAPK signaling complex to drive ROS production in an S‐nitrosylation dependent manner. Molecular docking identifies several bioactive DDIT4‐inteacting compounds including imatinib, nilotinib, and nateglinide, all of which are confirmed to attenuate hepatic ROS production, dampen p38‐MAPK signaling, and ameliorate liver injury by influencing DDIT4 S‐nitrosylation. Importantly, positive correlation between ROS levels and BRG1/DDIT4/S‐nitrosylated DDIT4 levels is detected in human liver biopsy specimens. In conclusion, the data reveal a transcription‐based signaling cascade that contributes to ROS production in liver injury.

## Graphical abstract

Brahma‐related gene 1 interacts with hypoxia inducible factor‐1*α* to activate the transcription of DNA damage‐induced transcript 4 (DDIT4) in hepatocytes in response to injurious stimuli. DDIT4 undergoes S‐nitrosylation at a conserved cysteine residue, which licenses DDIT4 to be incorporated into the p38‐MAPK signaling complex. The ensuring p38 activation leads to accelerated reactive oxygen species production and liver injury.

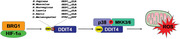

## 1 Introduction

Reactive oxygen species (ROS) wields bifurcated influences on hepatic homeostasis. Deemed essential for fetal liver development^[^ ^1^ ^]^ and postinjury liver regeneration in adults,^[^ ^2^ ^]^ excessive ROS production and accumulation is frequently observed in and blamed as a culprit for a host of liver diseases.^[^ ^3^ ^]^ Intracellular redox status is programmed by an intricate web of signaling molecules and nuclear transcription factors. Mitogen‐activated protein kinase (MAPK), subcategorized into the p38 pathway, the JNK pathway, and the ERK pathway, represents a structurally and functionally divergent group of proteins residing in the center of the intracellular ROS signaling network.^[^ ^4^ ^]^ In quiescent cells MAPKs are unphosphorylated and thus inactive; cued by extracellular/intracellular injurious stimuli, MAPKs become phosphorylated thus acquiring the ability to relay the ROS signaling. MAPKs typically function within a signaling complex glued together by scaffolding proteins.^[^ ^5^ ^]^ For instance, several different scaffolding proteins including TAB1,^[^ ^6^ ^]^ RACK1,^[^ ^7^ ^]^ and JIP4^[^ ^8^ ^]^ have been identified to activate p38‐MAPK signaling in a cell type‐ and environment‐dependent manner. The role of MAPK scaffolding proteins in hepatic ROS production is not completely understood.

DNA damage‐induced transcript 4 (DDIT4), variously termed REDD1 or RTP801, was initially identified as a developmentally regulated target of p53/p63 that drives epithelial cell differentiation by fueling ROS production.^[^ ^9^ ^]^ Considered as a stress response protein, DDIT4 is present at low levels in most somatic cells including hepatocytes but can be upregulated by a number of stimuli at the transcriptional level. Recent studies have clearly implicated DDIT4 in the regulation of redox status. For instance, it has been shown that DDIT4 forms a complex with the pro‐oxidant protein TXNIP to maintain cellular ROS production and adaptation to energy demand by controlling autophagic flux.^[^ ^10^ ^]^ In addition, Qiao et al. have proposed that DDIT4 deficiency results in oncogenic transformation by augmenting ROS‐cleansing NADPH to skew cellular metabolism.^[^ ^11^ ^]^ On the contrary, DDIT4 can also promote the pathogenesis of diabetic retinopathy by mediating ROS‐induced degradation of the antioxidant transcription factor NRF2.^[^ ^12^ ^]^ Currently, there is insufficient evidence regarding the transcriptional regulation of DDIT4 during liver injury or its pathophysiological implication.

Mammalian transcriptional regulation is intimately coupled to the epigenetic machinery. Brahma‐related gene 1 (BRG1) is the core component of the SWI/SNF chromatin remodeling complex. We have previously reported that hepatocyte conditional BRG1 knockout (LKO) mice exhibited deceleration of ROS accumulation in the liver compared to the WT littermates in a model of non‐alcoholic steatohepatitis.^[^ ^13^ ^]^ In the present study we have identified DDIT4 as a novel target for BRG1, which undergoes cysteine S‐nitrosylation and scaffolds the p38 signaling complex to promote ROS‐induced liver injury. More importantly, we demonstrate that the clinically available drugs Nateglinide, Imatinib, and Nilotinib can mitigate liver injury by targeting DDIT4 S‐nitrosylation to dampen MPAK signaling and hepatic ROS production.

## 2 Results

### 2.1 BRG1 Is Universally Required for Hepatic ROS Generation

To investigate the universal requirement for BRG1 in the regulation of hepatic redox status during liver injury, hepatocyte conditional BRG1 knockout (LKO) mice and wild type mice were compared in three different animal models. In MCD diet induced steatotic liver injury, bile duct ligation (BDL) surgery induced cholestatic liver injury, and in CCl_4_ injection induced hepatotoxic injury, BRG1 deficiency in hepatocytes invariably dampened hepatic ROS production (Figure S1, Supporting Information). Next, we examined the effect of BRG1 deficiency on ROS production in cultured hepatocytes. Two pathologically relevant stimuli, palmitate (PA) and bile acid (BA), instigated ROS production in hepatocytes; BRG1 knockdown by siRNAs or inhibition by a small‐molecule compound (PFI‐3) suppressed ROS production (Figure S2, Supporting Information). In accordance, primary hepatocytes isolated from the BRG1 LKO mice produced less ROS than those isolated from the WT mice when stimulated with PA or BA (Figure S2, Supporting Information).

### 2.2 PCR‐Array Screening Identifies BRG1 Target Genes Related to Redox Regulation

**Figure 1:**
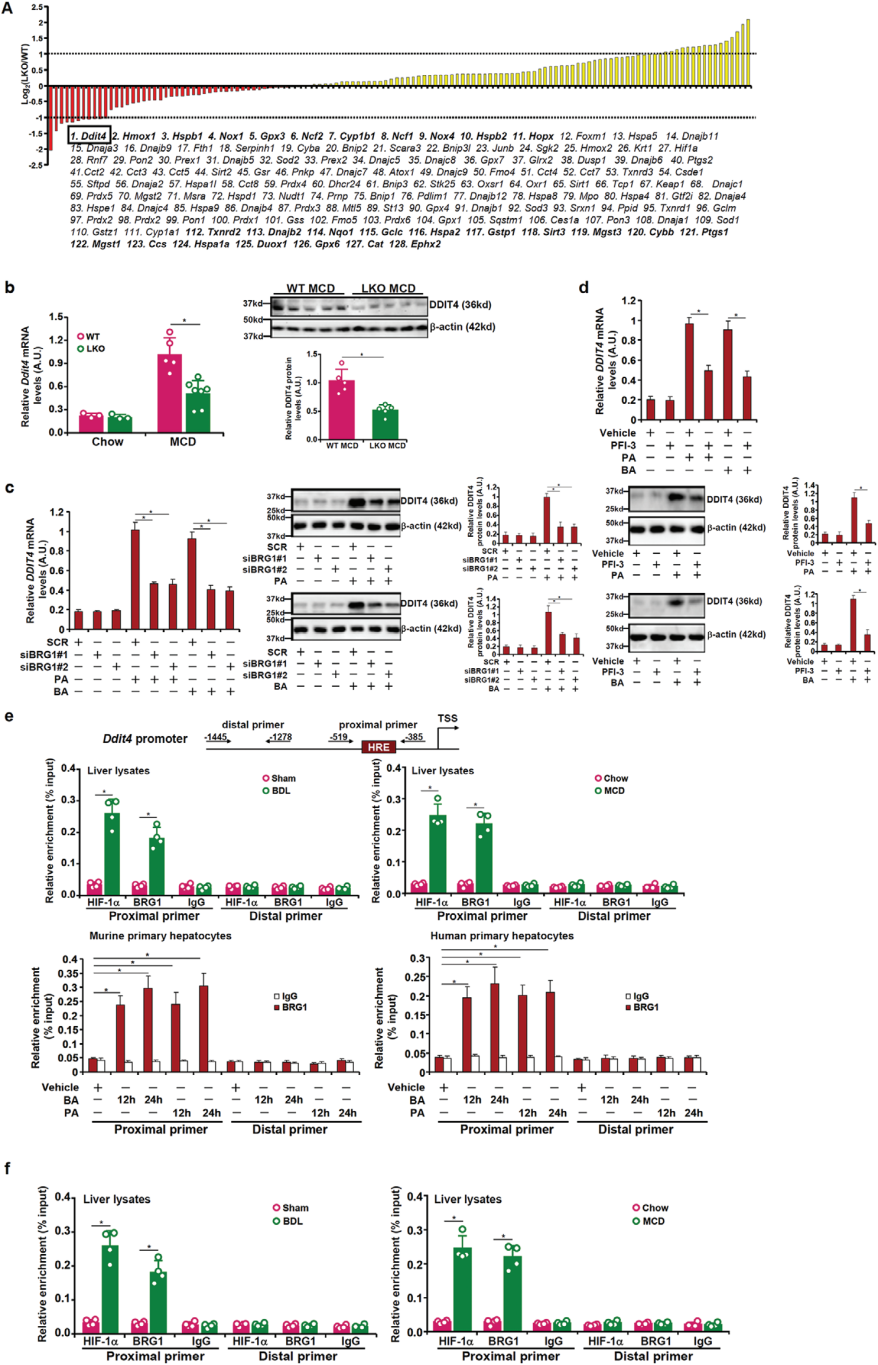
BRG1 regulates DDIT4 expression in vivo and in vitro. a) WT and BRG1 LKO mice were fed an MCD diet for 4 weeks. PCR‐array was performed as described in the Experimental Section. Genes with over twofold changes are highlighted in bold. b) BRG1 LKO and WT mice were fed an MCD diet for 4 weeks as described in the Experimental Section. DDIT4 expression was examined by qPCR and Western. c) Primary human hepatocytes were transfected with siRNA targeting BRG1 or scrambled siRNA (SCR) followed by treatment with PA (0.4 × 10^−3^ m) or BA (0.5 × 10^−3^ m) for 24 h. DDIT4 expression was examined by qPCR and Western. d) Primary human hepatocytes were treated with PA (0.4 × 10^−3^ m) or BA (0.5 × 10^−3^ m) in the presence or absence of PFI‐3 (5 × 10^−6^ m) for 24 h. DDIT4 expression was examined by qPCR and Western. e) (Upper panel) C57 mice were fed an MCD diet for 4 weeks or subjected to the BDL surgery for 2 weeks. (Bottom panel) Primary human or murine hepatocytes were treated with PA (0.4 × 10^−3^ m) or BA (0.5 × 10^−3^ m) and harvested at indicated time points. ChIP assays were performed with anti‐BRG1 or IgG. f) C57 mice were fed an MCD diet for 4 weeks or subjected to the BDL surgery for 2 weeks. Re‐ChIP assay was performed with indicated antibodies. *N* = 4 mice for each group.

In order to identify potential transcriptional targets for BRG1 involved in the regulation of cellular redox status, we performed a PCR‐array based screening. Relative expression levels of 128 redox‐related genes were profiled in liver samples isolated from WT mice and LKO mice fed on an MCD diet for 4 weeks; a minimal change of twofold was used as cut‐off. As shown in **Figure** **1**a, 28 genes met this criterion: 11 genes were downregulated in the LKO livers compared to the WT livers by more than twofold (equivalent to a log_2_ΔΔct values below −1) and 17 genes were upregulated in the LKO livers compared to the WT livers by more than twofold (equivalent to a log_2_ΔΔct values above +1). Considering that BRG1 is mostly a transcriptional activator, one would expect a primary target of BRG1 to be downregulated by BRG1 deficiency. Based on these criteria, 11 genes were IDed as putative BRG1 target genes. We focused on DDIT4, the gene most sensitive to BRG1 deletion, for the remainder of the study.

### 2.3 BRG1 Regulates DDIT4 Expression In Vivo and In Vitro

We next verified whether DDIT4 expression could be regulated by BRG1 in different animal and cell culture models. DDIT4 expression was robustly induced in the liver by MCD diet feeding (Figure 1b), by the BDL procedure (Figure S3, Supporting Information), and by CCl_4_ injection (Figure S4, Supporting Information); the induction of DDIT4 expression by the injurious stimuli was dampened by BRG1 deficiency. In human primary hepatocytes (Figure 1c) and HepG2 cells (Figures S5 and S6, Supporting Information), treatment with PA or BA markedly upregulated DDIT4 expression whereas BRG1 knockdown appreciably attenuated DDIT4 upregulation. Similarly, BRG1 inhibition ameliorated DDIT induction (Figure 1d; Figures S5 and S6, Supporting Information). Of note, BRG1 knockdown or inhibition had a minimal impact on DDIT4 levels without PA/BA treatments suggesting that BRG1 might not be essential for maintaining basal DDIT4 expression. Finally, primary hepatocytes isolated from WT mice responded better to PA or BA treatment in terms of DDIT4 induction than those isolated from BRG1 LKO mice (Figure S7, Supporting Information). Together, these data confirm that BRG1 is essential for stress‐induced DDIT4 expression in hepatocytes both in vivo and in vitro.

### 2.4 BRG1 Directly Activates DDIT4 Transcription

The following experiments were performed to determine whether or not BRG1 could activate DDIT4 expression at the transcriptional level. Exposure to PA treatment stimulated the DIT4 promoter–reporter activity, which was further augmented by BRG1 overexpression (Figure S8, Supporting Information). Serial deletions introduced to the DDIT4 promoter–reporter construct did not significantly alter its activation unless and until the deletion extended beyond −500 relative to the transcription start site (Figure S8, Supporting Information). A close examination of the DDIT4 sequences between −500 and −150 revealed a conserved binding motif for hypoxia inducible factor (HIF) (Figure S8, left panel, Supporting Information). Indeed, mutagenesis of the HIF binding site abrogated induction of the DDIT4 promoter (Figure S8, right panel, Supporting Information). Chromatin immunoprecipitation (ChIP) assay performed in liver lysates and primary human/murine hepatocytes showed that binding of BRG1 to the DDIT4 promoter was greatly increased by stimuli (Figure 1e). Co‐immunoprecipitation showed that BRG1 and HIF‐1*α* interacted with each other in the murine livers (Figure S9, Supporting Information). Re‐ChIP assay confirmed the formation of a BRG1‐HIF‐1*α* complex on the DDIT4 promoter in the injured livers (Figure 1f) and in PA/BA‐treated hepatocytes (Figure S10, Supporting Information).

### 2.5 DDIT4 Knockdown Attenuates ROS Production

**Figure 2:**
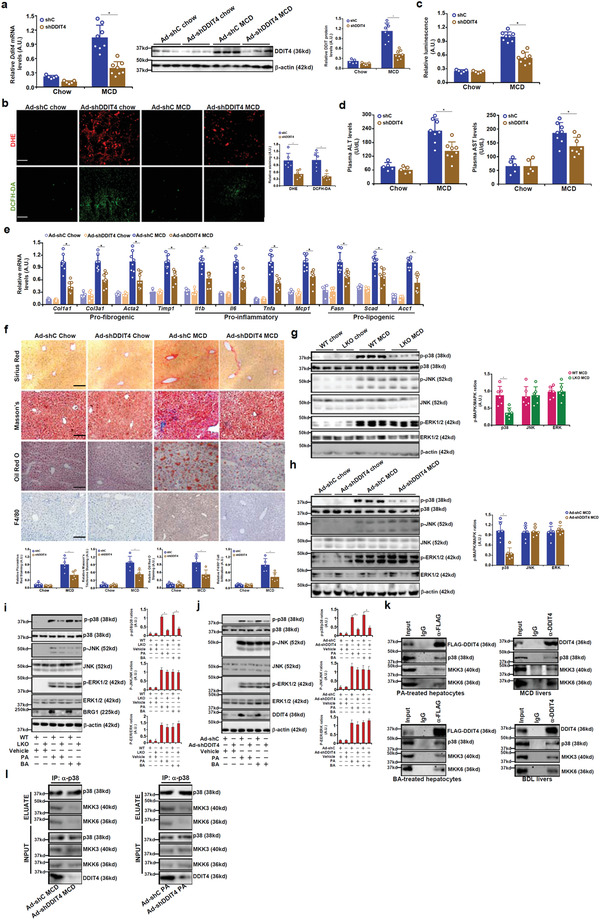
DDIT4 modulates hepatic ROS production by promoting MAPK‐p38 signaling complex assembly. C57/B6 mice were injected with adenovirus carrying DDIT4 shRNA or control adenovirus followed by MCD feeding for 4 weeks. a) DDIT4 expression levels were verified by qPCR and Western. b) Frozen sections were stained with DHE or DFHC. c) ROS levels in the liver homogenates were measured with a fluorimetric kit. d) Plasma ALT and AST levels. e) Gene expression levels were examined by qPCR. f) Liver sections were stained with picrosirius red, Masson's trichrome, anti‐F4/80, and oil red O. *N* = 5–8 mice for each group. g) BRG1 LKO and WT mice were fed an MCD diet for 4 weeks. Protein levels were examined in liver lysates by Western. h) C57/B6 mice were injected with adenovirus carrying DDIT4 shRNA or control adenovirus followed by MCD feeding for 4 weeks. Protein levels were examined in liver lysates by Western. i) Primary murine hepatocytes isolated from BRG1 LKO and WT mice were treated with PA (0.4 × 10^−3^ m) or BA (0.5 × 10^−3^ m) for 12 h. Protein levels were examined in cell lysates by Western. j) Primary murine hepatocytes were infected with Ad‐shC or Ad‐shDDIT4 followed by treatment with PA (0.4 × 10^−3^ m) or BA (0.5 × 10^−3^ m) for 12 h. Protein levels were examined in cell lysates by Western. k) (Left panel) Primary murine hepatocytes were infected with Ad‐FLAG‐DDIT4 followed by treatment with PA (0.4 × 10^−3^ m) for 12 h. IP was performed with anti‐FLAG. (Right panel) C57/BL mice were fed an MCD diet for 4 weeks. IP was performed with anti‐DDIT4. l) C57/B6 mice were injected with adenovirus carrying DDIT4 shRNA or control adenovirus followed by MCD feeding for 4 weeks. IP was performed with anti‐p38.

We next addressed the following issue: whether DDIT4 is indispensable for ROS production in liver injury. First, we assessed the effect of DDIT4 knockdown, mediated by adenoviral delivery of shRNA, on hepatic ROS production in the MCD model and the BDL model. Marked downregulation of hepatic DDIT4 expression was detected in mice injected with Ad‐shDDIT4 compared to those injected with Ad‐shC (**Figure** **2**a) in the MCD‐fed mice. Consequently, hepatic ROS levels were decreased as determined by DHE/DCFH‐DA staining (Figure 2b) and luminescence measurement (Figure 2c). Accompanying ROS downregulation DDIT4 knockdown attenuated steatotic liver injury as evidenced by plasma ALT and AST levels (Figure 2d), quantitative PCR analysis of genes involved in pro‐fibrogenic, pro‐inflammatory, and pro‐lipogenic responses (Figure 2e), picrosirius red/Masson's trichrome staining of ECM deposition, oil red O staining of hepatic lipid droplets, and F4/80 staining of macrophage infiltration (Figure 2f). In addition, quantification of liver triglyceride, cholesterol, and hydroxylproline contents all pointed to amelioration of steatosis as a result of DDIT4 knockdown (Figure S11, Supporting Information).

In the BDL model of liver injury, DDIT4 knockdown similarly dampened ROS production, mitigated liver injury, weakened hepatic inflammation, and assuaged liver fibrosis (Figure S12, Supporting Information). In cultured hepatocytes, DDIT4 depletion ameliorated ROS production induced by PA or BA treatment (Figure S13, Supporting Information).

### 2.6 DDIT4 Is Essential for the Assembly of the p38‐MAPK Signaling Complex

The cellular redox status is regulated by the MAPK signaling pathway.^[^ ^4^ ^]^ Deficiency in either BRG1 or DDIT4 in the liver suppressed p38‐MAPK phosphorylation without altering either JNK or ERK phosphorylation in the MCD model (Figure 2g,h) and the BDL model (Figure S14, Supporting Information). Similarly, BRG1 deletion (Figure 2i) or DDIT4 depletion (Figure 2j) in primary hepatocytes attenuated PA‐induced or BA‐induced p38 phosphorylation while leaving JNK/ERK phosphorylation intact. Basal MAPK activities were not affected by either BRG1 deletion or DDIT4 depletion. Adding to the evidence that the BRG1‐DDIT4 axis likely contributes to ROS production is the observation that administration of a p38‐sepcific inhibitor, but neither an ERK‐specific inhibitor nor a JNK‐specific inhibitor, blocked the restoration of ROS levels by exogenous DDIT4 in BRG1‐null hepatocytes (Figure S15, Supporting Information).

DDIT4 is primarily located in the cytoplasm.^[^ ^9^ ^]^ We therefore hypothesized that DDIT4 might function as a scaffold protein coordinating the assembly of the p38 signaling complex. Co‐immunoprecipitation assays performed in murine primary hepatocytes and in murine livers demonstrated that DDIT4 could be detected in the p38 signaling complex that includes p38, MKK3, and MKK6 (Figure 2k). GST pull‐down (Figure S16A, Supporting Information) and fluorescence resonance energy transfer (FRET) assay (Figure S16B, Supporting Information) indicated that DDIT4 might directly interact with p38, but neither MKK3 nor MKK6. In addition, mutagenesis studies appeared to suggest that the middle part of DDIT4 (76–160) was sufficient to mediate its interaction with p38 whereas neither the N‐terminus nor the C‐terminus was indispensable for p38 interaction (Figure S17, Supporting Information). Of interest, DDIT4 depletion disrupted the assembly of the p38 complex as evidenced by weakened interaction between p38 and MKK3/6 (Figure 2l). To determine the stoichiometry of the p38‐DDIT4 interaction, isothermal calorimetry assay was performed. The p38/DDIT4 molar ratio, based on the titration data, was calculated to be 0.91 ± 0.11, pointing to a 1:1 molar binding stoichiometry (Figure S18, Supporting Information).

### 2.7 DDIT4 S‐Nitrosylation Promotes the Assembly of the p38 Signaling Complex

**Figure 3:**
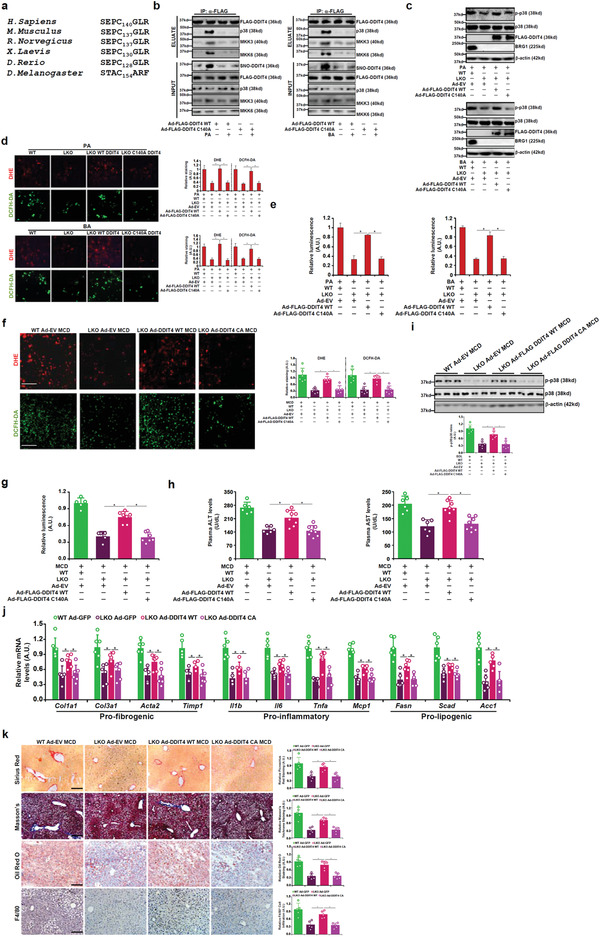
S‐nitrosylation of DDIT4 promotes assembly of p38 signaling complex. a) Cross‐species comparison of DDIT4 protein sequences. The conserved SNO site (C) is highlighted. b) Primary murine hepatocytes were infected with adenovirus carrying DDIT4 expression constructs followed by treatments with PA or BA. IP was performed with anti‐FLAG. DDIT4 S‐nitrosylation was examined by biotin exchange followed by Western. c–e) Primary hepatocytes isolated from WT and BRG1 LKO mice were infected with indicated adenovirus followed by treatment with PA or BA. MAPK phosphorylation was examined by Western. ROS levels were examined by DHE staining, DCFH‐DA staining, or a fluorimetric kit. f–k) BRG1 LKO mice were injected via tail vein Ad‐FLAG‐DDIT4 or Ad‐EV followed by MCD feeding along with the WT mice for 4 weeks. Frozen sections were stained with DHE or DFHC. ROS levels in the liver homogenates were measured with a fluorimetric kit. Plasma ALT and AST levels. MAPK phosphorylation was examined by Western. Gene expression levels were examined by qPCR. Liver sections were stained with picrosirius red, Masson's trichrome, anti‐F4/80, and oil red O. *N* = 6–8 mice for each group.

Mounting evidence indicates that protein S‐nitrosylation represents a new paradigm in ROS signaling.^[^ ^14^ ^]^ Of note, S‐nitrosylation of DDIT4 was strikingly upregulated in the injured livers (Figure S19, Supporting Information) and in PA or BA treated hepatocytes (Figure S20, Supporting Information). Bioinformatic analysis aided by a prediction tool^[^ ^15^ ^]^ revealed a cysteine residue (C140), which fell within the part of DDIT4 that mediates its interaction with p38, putatively subject to S‐nitrosylation is conserved across several different species (**Figure** **3**a). To verify whether this cysteine residue represents the major S‐nitrosylation site within DDIT4, primary hepatocytes were infected with adenovirus carrying either FLAG‐tagged WT or mutant DDIT4 in which the cysteine residue is substituted by an alanine (C140A). Whereas treatment with PA or BA markedly augmented S‐nitrosylation of WT DDIT4, virtually no change in S‐nitrosylation was detected for the mutant DDIT4 (Figure 3b). This observation was further confirmed by GST pull‐down assay and FRET assay (Figure S21, Supporting Information)

We then assessed the functional relevance of DDIT4 S‐nitrosylation to p38 signaling and ROS production in hepatocytes. Unlike the WT DDIT4, which became incorporated into the p38 complex upon PA/BA stimulation, the C140A mutant failed to interact with the p38 signaling complex (Figure 3c). As a result, the C140A mutant, when over‐expressed in BRG1 deficient hepatocytes, was unable to restore p38 phosphorylation (Figure 3c) and ROS production (Figure 3d,e). Taken together, these data suggest that S‐nitrosylation is essential for DDIT4 to regulate ROS production likely by enabling DDIT4 to scaffold the p38 complex.

### 2.8 S‐Nitrosylation Enables DDIT4 to Rescue ROS Production in BRG1‐Deficient Mice

We asked whether exogenous DDIT4 could normalize ROS production in BRG1 LKO mice. Forced expression of WT and C140A DDIT4 in the liver was achieved by tail vein injection of adenovirus carrying respective vectors; the mice were then fed an MCD diet for 4 weeks. As shown in Figure S21 of the Supporting Information, injection of the adenovirus carrying the DDIT4 construct more than compensated the loss of DDIT4 expression in the LKO mice compared to the WT mice injected with the control adenovirus. Importantly, reintroduction of WT DDIT4, but not the C140A DDIT4, in the LKO livers increased ROS accumulation, bringing it up to the level observed in the WT livers (Figure 3f,g). Of note, the severity of steatotic injury, as measured by plasma ALT and AST levels, was ameliorated in the LKO livers and forced expression of WT DDIT4, but not C140A DDIT4, reignited the injury (Figure 3h). These observations were consistent with the changes in p38 phosphorylation: WT DDIT4, but not C140A DDIT4, restored p38 phosphorylation in the LKO livers (Figure 3i). QPCR analysis (Figure 3j), histological stainings (Figure 3k), and biochemical quantifications (Figure S22, Supporting Information) all pointed to the conclusion that S‐nitrosylation of DDIT4 might be essential for the solicitation of liver injury.

In a second model of BDL‐induced liver injury, DDIT4 overexpression overcame BRG1 deficiency to normalize ROS production, liver injury, and p38 phosphorylation in the LKO mice, all of which strictly relied on the intactness of cysteine 140 (Figure S23, Supporting Information).

### 2.9 Imatinib, Nilotinib, and Nateglinide Influence ROS Production and p38‐MAPK Signaling by Targeting DDIT4 S‐Nitrosylation

**Figure 4:**
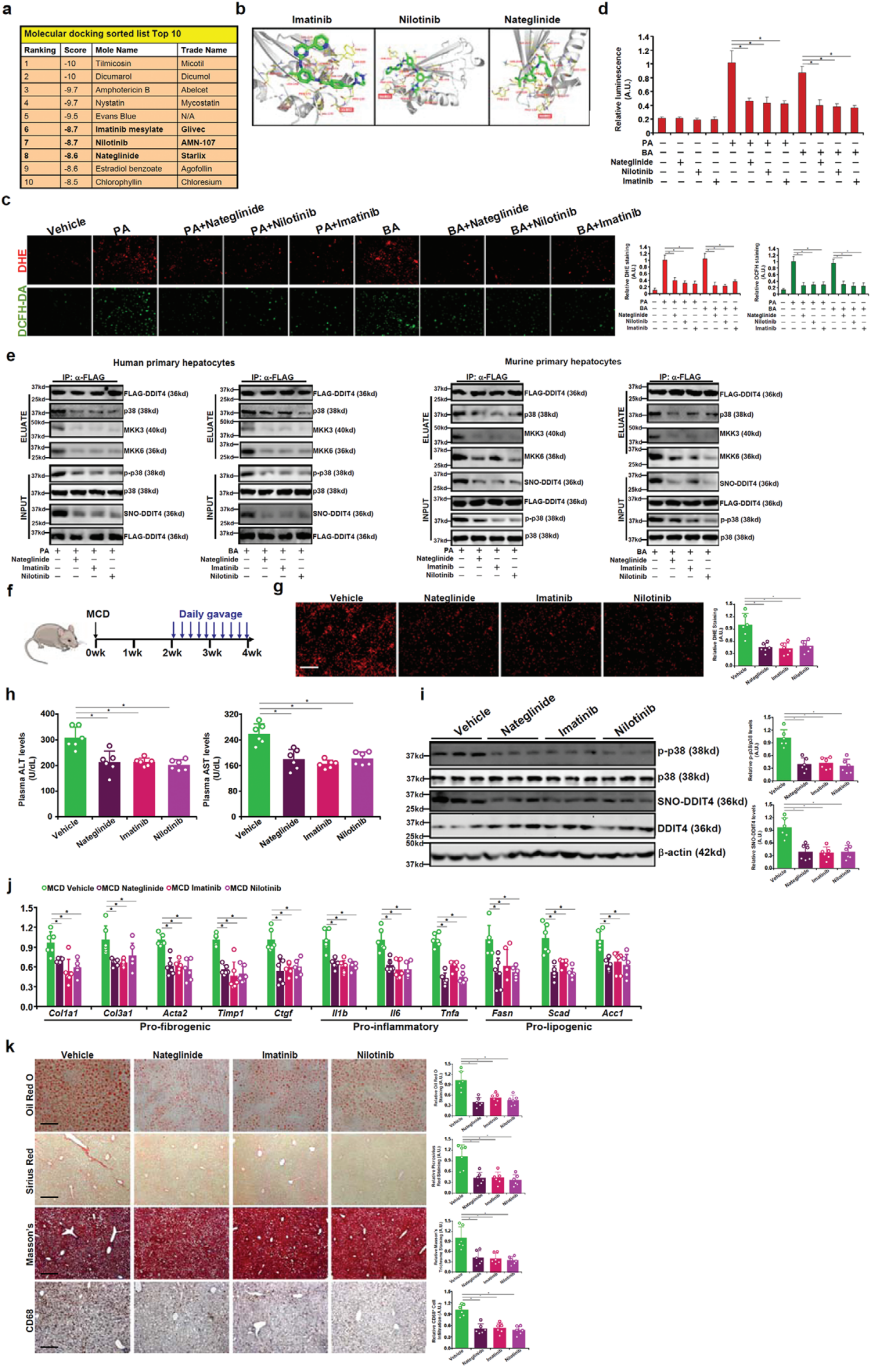
Imatinib, Nilotinib, and Nateglinide modulate ROS production and p38‐MAPK signaling by influencing DDIT4 S‐nitrosylation in vitro. a) List of top 10 hits. b) Scheme of molecular docking. The S‐nitrosylation site of DDIT4 (C140) is boxed. c,d) Human primary hepatocytes were treated with PA (0.4 × 10^−3^ m) or BA (0.5 × 10^−3^ m) in the presence or absence of indicated compounds. ROS levels were examined by DHE staining, DCFH‐DA staining, or a fluorimetric kit. e) Human primary hepatocytes were infected with adenovirus carrying DDIT4 expression constructs followed by treatments with PA or BA. IP was performed with anti‐FLAG. DDIT4 S‐nitrosylation was examined by biotin exchange followed by Western. f) Schematic protocol. g) Frozen sections were stained with DHE. h) Plasma ALT and AST levels. i) MAPK phosphorylation was examined by Western. j) Gene expression levels were examined by qPCR. k) Liver sections were stained with picrosirius red, Masson's trichrome, anti‐CD68, and oil red O. *N* = 6 mice for each group.

To explore the possibility of targeting DDIT4 in the intervention of liver injury, molecular docking was performed to screen the FDA approved drug database (**Figure** **4**a). Using ROS suppression as a criterion, we further narrowed the top 10 potential DDIT4 antagonists down to 3: Imatinib, Nilotinib, and Nateglinide (Figure S24, Supporting Information, and data not shown). All three compounds can potentially make contacts with DDIT4 in close proximity to the C140 residue (Figure 4b). The ability of these three compounds to inhibit PA/BA‐induced ROS production was confirmed in human primary hepatocytes (Figure 4c,d). Moreover, treatment with Imatinib, Nilotinib, or Nateglinide comparably suppressed DDIT4 S‐nitrosylation, disrupted the p38‐MAPK signaling complex and inhibited p38 phosphorylation in human and murine primary hepatocytes (Figure 4e).

Next, we evaluated the in vivo effects of the DDIT4‐targeting compounds (Figure 4f). All three compounds attenuated hepatic ROS levels (Figure 4g) and liver injury as judged by plasma ALT/AST levels (Figure 4h). DDIT4 S‐nitrosylation and p38‐MAPK phosphorylation levels were inhibited to equivalent extent by the three compounds (Figure 4i). Quantitative PCR (Figure 4j) and histological stainings (Figure 4k) confirmed that hepatic inflammation, fibrosis, and lipid deposition were all ameliorated by the DDIT4‐targeting compounds. Similar observations were made a second model of BDL‐induced cholestatic injury (Figure S25, Supporting Information). Of note, administration of these compounds to normal C57B6/L mice under physiological conditions did not appear to elicit any adversarial effects as judged by plasma aminotranferase activities (Figure S26A, Supporting Information), gross hepatic morphology (Figure S26B, Supporting Information, H&E staining), hepatic ROS levels (Figure S26B, Supporting Information, DHE staining), hepatic lipid droplet accumulation (Figure S26B, Supporting Information, oil red O staining), hepatic macrophage accumulation (Figure S26B, Supporting Information, CD68 staining), and hepatic fibrogenesis (Figure S26B, Supporting Information, Sirius Red/Masson's stainings). QPCR confirmed that administration of these compounds was not associated with significant changes in hepatic expression of proinflammatory/prolipogenic/profibrogenic mediators (Figure S26C, Supporting Information). Western blotting showed that basal MAPK activities (phosphorylation levels) were not influenced by Imatinib/Nilotinib/Nateglinide administration (Figure S25D, Supporting Information). These data, when taken together, are compatible with the notion that these compounds do not interfere with normal hepatic functions.

### 2.10 Expression Levels of BRG1 and DDIT4 Correlate with ROS Levels in Humans

**Figure 5:**
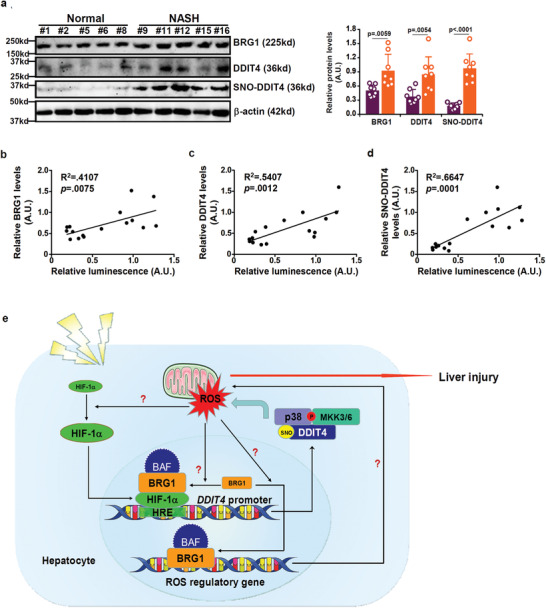
Expression levels of BRG1 and DDIT4 correlate with ROS levels in human NASH liver biopsy specimens. a) Representative Western blots of proteins in NASH and control livers. b–d) Linear regression was performed by Graphpad Prism. *N* = 8 for each group. e) A schematic model.

We finally evaluated the validity of our working model that BRG1‐induced DDIT4 contributes to ROS accumulation via S‐nitrosylation dependent assembly of p38 signaling complex in human specimens. Compared to the healthy individuals, expression levels of BRG1 and DDIT4 were remarkably augmented in the livers of patients diagnosed with nonalcoholic steatohepatitis (NASH). In addition, higher levels of DDIT4 S‐nitrosylation were detected in the NASH livers than in the control livers (**Figure** **5**a). More important, a positive correlation was identified between ROS levels and BRG1/DDIT4/DDIT4 S‐nitrosylation levels (Figure 5b–d).

## 3 Discussion

ROS‐fueled damages are considered as a major driving force of liver injury. Here we detail a novel transcription‐based signaling cascade in which the chromatin remodeling protein BRG1 induces the expression of DDIT4, which in turn promotes the assembly of the p38‐MAPK signaling complex and ROS production in hepatocytes to instigate liver injury (Figure 5e).

DDIT4 has been reported to exert versatile effects on a wide range of pathophysiological events including ROS production by interacting with other proteins. Previously Dennis et al. have shown that DDIT4 bridges PP2A‐mediated dephosphorylation of Akt to inhibit mTOR signaling in response to nutrient influx.^[^ ^16^ ^]^ In addition, Michel et al.^[^ ^17^ ^]^ have reported that Ca^2+^/calmodulin recruitment of DDIT4 to the plasma membrane facilitates signaling transduction downstream of several different G‐protein coupled receptors. Our data show that DDIT4 regulates hepatic ROS production likely via scaffolding the p38 signaling complex that includes p38, MKK3, and MKK6. These data collectively seem to suggest that functioning as a signaling module may indeed represent the primary mode of function for DDIT4. On the other hand, the present model as proposed should be interpreted with precaution because it is unlikely to encapsulate the complex nature of DDIT4‐mediated liver injury in its entirety. A recent study by Giorgetti‐Peraldi and colleagues argues that DDIT4 may contribute to steatotic liver injury in mice at least partly by promoting de novo lipogenesis.^[^ ^18^ ^]^ It is equally unfathomable that stabilization of the p38 signaling complex may be the sole mechanism underlying DDIT4‐dependent ROS production in hepatocytes. Many of the previously characterized DDIT4‐regulated signaling pathways are noted for their roles in ROS production and can form extensive dialogues with the MAPK pathway.^[^ ^19^ ^]^ The issue is further compounded by the observations that hepatocyte‐specific p38*α* deletion during embryogenesis predisposes the mice to the development of hepatocellular carcinoma likely owing to aberrant ROS accumulation where p38*α* deletion in mature hepatocytes dampens ROS accumulation and protects the mice from liver injury;^[^ ^20^, ^21^ ^]^ the contributions of the three other p38 isoforms to hepatic redox regulation are currently ambiguous. A comprehensive profiling of DDIT4‐dependent pro‐ROS signalome will hopefully provide more mechanistic insight on its role as a regulator of liver injury.

We report here that the ability of DDIT4 to bridge the p38 complex formation is strictly reliant on its S‐nitrosylation. Several reports have highlighted the influence of post‐translational modifications on DDIT4 function. Katiyar et al. have shown that two N‐terminal threonine residues (T23/T25) of DDIT4 are subjected to GSK3‐mediated phosphorylation, which then serve as a degron for proteasomal targeting and eventual degradation.^[^ ^22^ ^]^ DDIT4 can also be modified by K63‐conjugated ubiquitination and subsequent degradation, through two different E3 ligases Parkin and NEDD4, in neurons.^[^ ^23^, ^24^ ^]^ Part of the 3D structure for DDIT4, including the C140 residue subjected to S‐nitrosylation, has been delineated,^[^ ^25^ ^]^ which unfortunately offers little clue as to how the DDIT4‐p38 interaction is achieved likely owing to the fact it does not contain to any known functional domains. Therefore, it is not immediately clear how this modification could alter the DDIT4 conformation and facilitate its interaction with the p38 signaling complex. Of note, substitution of C140 with a serine impaired its function as an inhibitor of mTOR signaling^[^ ^25^ ^]^ suggesting that this region may be critical for brokering the interaction of DDIT4 with other factors and thus an attractive target for molecular modeling when screening for potential small‐molecule DDIT4 agonists/antagonists. Cysteine S‐nitrosylation, in addition to altering protein–protein interactions, modulates intracellular localization and half‐life of target proteins.^[^ ^26^ ^]^ Whereas overall DDIT4 protein level appeared to be unaffected by its S‐nitrosylation status, it remains to be determined whether S‐nitrosylation can target DDIT4, a primarily cytosol‐residing protein, to other subcellular compartments and what, if so, the pathophysiological relevance of its trans‐location may be. Furthermore, a proteomic analysis of S‐nitrosylation‐dependent DDIT4 interacome in hepatocytes may shed additional light on the mechanism whereby DDIT4 contributes to ROS production during liver injury.

The most intriguing finding of the current report is perhaps the observation that three FDA‐approved drugs, Nilotinib, Imatinib, and Nateglinide, can potentially mitigate liver injury in two different animal models possibly by blocking DDIT4 S‐nitrosylation to dampen p38‐MAPK signaling (Figure 4).The effects of Nilotinib^[^ ^27^ ^]^ and Imatinib^[^ ^28^ ^]^ on liver injury have been reported recently, focusing on TGF‐*β* mediated activation of hepatic stellate cells and SREBP‐mediated lipogenesis, respectively. Because elevated ROS levels in the liver serve as a critical fuel for fibrogenesis^[^ ^29^ ^]^ and lipogenesis,^[^ ^30^ ^]^ our data certainly provide additional mechanistic insights into the mode of action for Nilotinib and Imatinib. Nateglinide, on the other hand, has not been considered as a potential therapeutic option for liver injuries thus far. A member of the meglitinide class, Nateglinide is used as an antidiabetic medication by promoting insulin release.^[^ ^31^ ^]^ The primary target for Nateglinide is considered to be ATP‐dependent K^+^ channels.^[^ ^32^ ^]^ Handling of the potassium channels seems to be intimately coupled to mitochondrial (dys)function including ROS generation^[^ ^33^ ^]^ but its connection to DDIT4 is not necessarily clear. Of note, KATP blockade in keratinocytes alleviates inflammation and ROS production by suppressing MAPK‐p38 signaling.^[^ ^34^ ^]^ Because we observed here that Nateglinide treatment in cultured hepatocytes was sufficient to disrupt p38‐MAPK signaling and dampen ROS production (Figure 4), the possibility that the beneficial effects of Nateglinide may rely on insulin release can be essentially ruled out although the exact mechanism underlying its protective effect deserves future attention. Nevertheless, our data incentivize further efforts to expand the scope of compounds for DDIT4‐based drug‐mining.

There are a few lingering issues that may deserve further attention. First, both BRG1 levels and HIF‐1*α* levels appear to be increased, either transcriptionally or post‐transcriptionally, by PA/BA treatment in hepatocytes suggesting that ROS production may be autoamplified by stimulating the expression of its regulators (e.g., BRG1 and HIF‐1*α*). Similarly, it is tempting to speculate that DDIT4, by virtue of promoting ROS production, may reciprocally contribute to the upregulation of BRG1 and HIF‐1*α* thus fueling its own induction. Second, although our data clearly demonstrate the presence of a BRG1‐HIF‐1*α* complex on the DDIT4 promoter, it is unclear whether this complex may contain additional factors. BRG1 typically functions in the context of BRG1/BRM‐associated factor (BAF) complex whose composition is often fluid.^[^ ^35^ ^]^ Murakami et al. have observed an interaction between HIF‐1*α* and BAF180 (also known as PBRM1) in renal carcinoma cells.^[^ ^36^ ^]^ On the other hand, He et al. have reported that BAF180 contributes to the maintenance of intestinal immune homeostasis by regulating ROS production.^[^ ^37^ ^]^ Whether these observations can be incorporated into our proposed model, i.e., a multiprotein complex binds to the DDIT4 promoter and activates DDIT4 transcription, is an open question. Additionally, although catalytic activity‐independent functions have been noted for BRG1,^[^ ^38^, ^39^ ^]^ it is not immediately clear whether the ATPase domain of BRG1 may be essential for its binding to the DDIT4 and/or for DDIT4 transactivation. Third, although DDIT4 S‐nitrosylation levels are drastically increased during liver injury the underlying regulatory mechanism has not been clearly defined. Generally, protein S‐nitrosylation results from increased intracellular nitric oxide (NO) levels catalyzed by NO synthases.^[^ ^26^ ^]^ Upregulation of iNOS, the predominant NOS isoform in hepatocytes, has been observed in multiple models of liver injury.^[^ ^40^, ^41^, ^42^ ^]^ Alternatively, cysteine S‐nitrosylation is removed by thioredoxin, which tends to decrease in the course of liver injury.^[^ ^43^, ^44^ ^]^ Therefore, it is possible that the observed changes in DDIT4 S‐nitrosylation could be attributed to a combination of increased donor (NO) bioavailability and reduced elimination. Fourth, the precise mechanism whereby DDIT4‐mediated assembly of the p38‐MKK3‐MKK6 complex leads to p38 activation remains to be ascertained. Canonical activation of p38 by MKK3/MKK6 requires the binding of MKK3/6 to multiple sites of p38 via identical motifs known as kinase interaction motifs (KIM).^[^ ^45^, ^46^ ^]^ It is possible that DDIT4 binding to p38 may induce conformational changes of p38 so that some contact sites of p38 (e.g., the hydrophobic groove) become more exposed to accommodate MKK3/MKK6 whereas other contact sites of p38 (e.g., the common docking site) become less exposed to block the access of phosphatases and subsequent dephosphorylation. Structural studies comparing the fine 3D crystallography data DDIT4‐bound p38 and DDIT4‐less p38 would shed additional light on this critical issue.

In summary, our data reinforce the role of BRG1 as a key regulator of hepatic homeostasis. Small inhibitors of BRG1 (e.g., PFI‐3) has been used in the preclinical studies of malignant cancers.^[^ ^47^ ^]^ More interestingly, we have uncovered DDIT4 as a direct downstream effector of BRG1 by coordinating the assembly of the p38‐MAPK signaling complex. Targeting the newly identified BRG1‐DDIT4 axis can be considered as a reasonable interventional strategy to treat liver injury.

## 4 Experimental Section

### Animals

All the animal experiments were reviewed and approved by the Nanjing Medical University Ethics Committee on Humane Treatment of Experimental Animals. Hepatocyte‐specific deletion of BRG1 was achieved by crossing the *Smarca4* ^f/f^ strain with the *Alb*‐Cre strain.^[^ ^48^ ^]^ To induce liver injury, 6–8 week‐old, male mice were subjected to BDL or the sham procedure and sacrificed 2 weeks after surgery as previously described.^[^ ^49^ ^]^ Alternatively, the mice were injected peritoneally with CCl_4_ (1.0 mL kg^−1^ body weight as 50%, vol/vol) or corn oil every other day for 2 weeks as previously described.^[^ ^50^ ^]^ In a third model of liver injury, the mice were fed an MCD diet for 4 weeks as previously described.^[^ ^51^ ^]^ In certain experiments, the mice were injected via tail vein adenovirus (1 × 10^9^ Pfu) carrying various expression constructs 2 weeks prior to the start of liver injury.

### Cell Culture, Plasmids, and Transient Transfection

Human hepatoma cells (HepG2) were maintained in DMEM supplemented with 10% fetal bovine serum (Hyclone). Primary murine hepatocytes were isolated and cultured as previously described.^[^ ^52^ ^]^ Primary human hepatocytes were purchased from Sigma. Human DDIT4 promoter–luciferase constructs^[^ ^53^ ^]^ and BRG1 expression constructs^[^ ^54^ ^]^ have been previously described. Small interfering RNAs were purchased from Dharmacon. Transient transfections were performed with Lipofectamine 2000. Luciferase activities were assayed 24–48 h after transfection using a luciferase reporter assay system (Promega).

### PCR Array

A customized PCR array (Qiagen) in a 384‐well format was performed to screen for BRG1 target genes essentially as previously described.^[^ ^55^ ^]^ 1 µg total RNA extracted from liver homogenates collected from MCD‐fed WT and BRG1 LKO mice was reverse‐transcribed using the RT^2^ First Strand kit supplied by the vendor. Then, the cDNA was mixed with 2× RT^2^ SYBR Green Mastermix and 25 µL of the mix was dispensed into the customized 384‐well plate that contained 128 preselected genes in duplicate plus 6 housekeeping genes for normalization. Quantitative PCR was performed on an Applied Biosystems StepOne Plus system. Cycle threshold (CT) values were calculated using StepOne software v2.1 with automatic baseline settings and a threshold of 1.2. The fold‐change for each gene was calculated using the ΔΔCT method and normalized by the housekeeping genes.

### RNA Isolation and Real‐Time PCR

RNA was extracted with the RNeasy RNA isolation kit (Qiagen) as previously described.^[^ ^56^, ^57^ ^]^ Reverse transcriptase reactions were performed using a SuperScript First‐strand Synthesis System (Invitrogen). Real‐time PCR reactions were performed on an ABI Prism 7500 system. The primers are listed in Table S1 of the Supporting Information. Ct values of target genes were normalized to the Ct values of house‐keeping control gene (18s, 5′‐CGCGGTTCTATTTTGTTGGT‐3′ and 5′‐TCGTCTTCGAAACTCCGACT‐3′ for both human and mouse genes) using the ΔΔCt method and expressed as relative mRNA expression levels compared to the control group which is arbitrarily set as 1.

### GST Pull‐Down Assay

Purified GST proteins were purchased from Cell Signaling Tech and mobilized to the Glutathione Agarose beads (Thermo Fisher) at 4 °C for 30 min. Cell lysates were then incubated with beads on a rotating platform at 4 °C for 2–4 h. The beads were washed three times with ice‐cold lysis buffer and bound proteins were eluted off by boiling in 1× SDS sample loading buffer for SDS‐PAGE electrophoresis and Western blotting.

### FRET Assay

FRET analysis was performed as previously described.^[^ ^58^ ^]^ Briefly, cell lysates containing Venus‐tagged DDIT4 (acceptor) were incubated with GST recombinant proteins (donor) in the presence of FRET reaction buffer (20 × 10^−3^ m Tris pH 7.0, 50 × 10^−3^ m NaCl, 0.01% NP‐40) in a 96‐well plate at room temperature for 1 h. Absorbance was measured on a spectrophotometer (Perkin Elmer) with the following settings: Ex: 337 nm, Em1: 520 nm, Em2: 486 nm. Data are expressed as the relative ratio of 520 nm absorbance and 486 nm absorbance.

### Protein Extraction and Western Blot

Whole cell lysates were obtained by resuspending cell pellets in RIPA buffer (50 × 10^−3^ m Tris pH 7.4, 150 × 10^−3^ m NaCl, 1% Triton X‐100) with freshly added protease and phosphatase inhibitors (Roche) as previously described.^[^ ^59^, ^60^ ^]^ Antibodies used for Western blotting are listed in Table S2 of the Supporting Information.

### ChIP

ChIP assays were performed essentially as described before.^[^ ^61^, ^62^, ^63^ ^]^ Chromatin was cross‐linked with 1% formaldehyde for 8 min room temperature, and then sequentially washed with ice‐cold phosphate‐buffered saline, Solution I (10 × 10^−3^ m HEPES, pH 7.5, 10 × 10^−3^ m EDTA, 0.5 × 10^−3^ m EGTA, 0.75% Triton X‐100), and Solution II (10 × 10^−3^ m HEPES, pH 7.5, 200 × 10^−3^ m NaCl, 1 × 10^−3^ m EDTA, 0.5 × 10^−3^ m EGTA). Cells were incubated in lysis buffer (150 × 10^−3^ m NaCl, 25 × 10^−3^ m Tris pH 7.5, 1% Triton X‐100, 0.1% SDS, 0.5% deoxycholate) supplemented with protease inhibitor tablet. DNA was fragmented into 500 bp pieces using a Branson 250 sonicator. Aliquots of lysates containing 100 µg of protein were used for each immunoprecipitation reaction with indicated antibodies followed by adsorption to protein A/G PLUS‐agarose beads (Santa Cruz Biotechnology). Precipitated DNA–protein complexes were washed sequentially with RIPA buffer (50 × 10^−3^ m Tris, pH 8.0, 150 × 10^−3^ m NaCl, 0.1% SDS, 0.5% deoxycholate, 1% Nonidet P‐40, 1 × 10^−3^ m EDTA), high salt buffer (50 × 10^−3^ m Tris, pH 8.0, 500 × 10^−3^ m NaCl, 0.1% SDS, 0.5% deoxycholate, 1% Nonidet P‐40, 1 × 10^−3^ m EDTA), LiCl buffer (50 × 10^−3^ m Tris, pH 8.0, 250 × 10^−3^ m LiCl, 0.1% SDS, 0.5% deoxycholate, 1% Nonidet P‐40, 1 × 10^−3^ m EDTA), and TE buffer (10 × 10^−3^ m Tris, 1 × 10^−3^ m EDTA pH 8.0), respectively. DNA–protein cross‐link was reversed by heating the samples to 65 °C overnight. Proteins were digested with proteinase K (Sigma), and DNA was phenol/chloroform‐extracted and precipitated by 100% ethanol. Precipitated genomic DNA was amplified by real‐time PCR using the primers listed in Table S1 of the Supporting Information. A total of 10% of the starting material is also included as the input.

### Biotin Switch Assay

S‐nitrosylation was detected by the biotin switch assay as previously described.^[^ ^64^ ^]^ Cells or liver tissues were resuspended in HENTS buffer (100 × 10^−3^ m Hepes, pH 7.4, 1 × 10^−3^ m EDTA, 0.1 × 10^−3^ m neocuproine, 0.1% SDS, and 1% Triton X‐100), mixed with blocking buffer (2.5% SDS, 10 × 10^−3^ m methyl methane thiosulfonate [MMTS] in HEN buffer [100 × 10^−3^ m HEPES, pH 7.4, 1 × 10^−3^ m EDTA, and 0.1 × 10^−3^ m Neocuproine]), and incubated for 20 min at 50 °C with frequent vortexing to block free thiol groups. After removal of excess MMTS by acetone precipitation, S‐nitrosothiols were reduced to thiols with 20 × 10^−3^ m ascorbate. Newly formed thiols were then linked with the sulfhydryl‐specific biotinylating reagent N‐[6‐biotinamido]‐hexyl]‐l′‐(2′pyridyldithio) propionamide (Biotin‐HPDP). Unreacted Biotin‐HPDP was removed by acetone precipitation, and the pellet was resuspended with HENS buffer (100 × 10^−3^ m HEPES, pH 7.4, 1 × 10^−3^ m EDTA, 0.1 × 10^−3^ m neocuproine, 1% SDS), neutralized, and centrifuged to clear undissolved debris. Biotinylated proteins were pulled down with Streptavidin‐agarose beads (Promega) and analyzed by Western blotting.

### DHE and DCFH‐DA Staining

Frozen liver sections or cells were stained with DHE (10 × 10^−6^ m) or DCFH‐DA (10 × 10^−6^ m) at 37 °C for 30 min. Fluorescence was visualized by cofocal microscopy (LSM 710, Zeiss). Quantifications were performed with ImageJ.

### Luminescence ROS Assay

Quantitative measurements of intracellular ROS were performed with a ROS‐Glo system (Promega). Briefly, a luminescence substrate solution was added to and incubated with cultured cells for 6 h followed by the addition of the diction solution. Luminescence was measured using a microplate reader. Data were expressed as relative ROS levels compared to the control group.

### Human NASH Biopsy Specimens

Liver biopsies were collected from patients with NASH referring to the First People's Hospital of Changzhou. Control liver samples were collected from donors without NASH but deemed unsuitable for transplantation. Written informed consent was obtained from subjects or families of liver donors. All procedures that involved human samples were approved by the Ethics Committee of the First People's Hospital of Changzhou and adhered to the principles outlined in the Declaration of Helsinki.

### Molecular Docking

A total of 1559 ligand molecules were obtained from the FDA approved drugs and molecules database (Topscience, Shanghai, China) and refined using the following protocol. The counterions, solvent moieties, and salts in the ligands were removed and the hydrogen atoms were added. The structures were chosen based on the MMFF94 force field using MOE (version 2010.10, Chemical computer group, Inc., Canada). Subsequently, the refined database was filtered using drug‐like analysis, liking Lipinski rules of five, and PAINS assay (http://cbligand.org/PAINS) before all molecules were automatically converted to PDBQT format. Open Babel software (http://openbabel.org/wiki/) and in‐house python script were used for manipulating the various chemical formats of ligand molecules. For optimization of docking simulations, the structure of DDIT4 (PDB code: 3lq9) was retrieved from RCSB protein data bank and prepared in three steps. First, all ions, crystalline water and native ligands were removed from the structure of DDIT4. Second, the missing hydrogen atoms were added. Lastly, the protein file was automatically converted to the PDBQT format. AutoDockVina was employed to screen the refined 1559 FDA library against DDIT4. The docking site was defined on glutathioneactive binding site (25.923, 3.873, and 18.582 Å) and the grid box was set as 50 × 50 × 50 Å in *x*, *y*, and *z* directions. During the docking process, the semiflexible docking simulations were performed employing Lamarckian genetic algorithm, and the receptor was kept rigid, while the ligands were flexible for rotation and exploration of the most probable binding conformations. After docking‐based virtual screening, the top 10 compounds with docking score were obtained for further analysis.

### Isothermal Calorimetry

Isothermal titration calorimetry was performed with a microcalorimeter (Malvern Panalytical) as previously described.^[^ ^65^ ^]^ FLAG‐DDIT4 was purified from primary hepatocytes by immunoprecipitation. GST‐p38 was dialyzed against the experimental buffer just prior to titration. Data were analyzed with an equilibrium binding model having a single equilibrium constant and enthalpy change.

### Statistical Analysis

For comparison between two groups, two‐tailed *t*‐test was performed. For comparison among three or more groups, one‐way ANOVA or two‐way ANOVA with post hoc Turkey analyses were performed using an SPSS package. The assumptions of normality were checked using Shapiro‐Wilks test and equal variance was checked using Levene's test; both were satisfied. *p* values smaller than 0.05 were considered statistically significant (*). All in vitro experiments were repeated at least three times and three replicates were estimated to provide 80% power.

## Conflict of Interest

The authors declare no conflict of interest.

## Author Contributions

Z.L., Q.Z., and Y.L. contributed equally to this work. Y.X. and Y.F.D. conceived the project. Z.L.L., Q.W.Z., Y.J.L., Y.X.Z., M.L., L.Y.L., X.M.C., and D.L.S. performed experiments and collected/analyzed data. Y.X. wrote the manuscript. Y.J.L., L.Y.L., and Y.X. provided funding.

## Supporting information

Supporting Information

## Data Availability

Research data are not shared.
