# Supplementary material for: DDIT4 S‐Nitrosylation Aids p38‐MAPK Signaling Complex Assembly to Promote Hepatic Reactive Oxygen Species Production
Source: Adv Sci (Weinh). 2021 Jul 26;8(18):2101957. doi: 10.1002/advs.202101957 (PMC8456271; doi:10.1002/advs.202101957)
Supplement: Supplementary file 1 — Supporting Information [file ADVS-8-2101957-s001.pdf]

© 2021 Wiley-VCH GmbH

ADVANCED SCIENCE
Open Access

Supporting Information

for *Adv. Sci.*, DOI: 10.1002/advs.202101957

DDIT4 S-Nitrosylation Aids p38-MAPK Signaling Complex Assembly to Promote Hepatic ROS Production

Zilong Li, Qianwen Zhao, Yunjie Lu, Yangxi Zhang, Luyang Li, Min Li, Xuemin Chen, Donglin Sun, Yunfei Duan, and Yong Xu**

Li ZL et al: DDIT4 S-nitrosylation aids p38-MAPK signaling complex assembly to promote hepatic ROS production
Online supplementary material
Supplementary Figures: 26
Supplementary Tables: 2

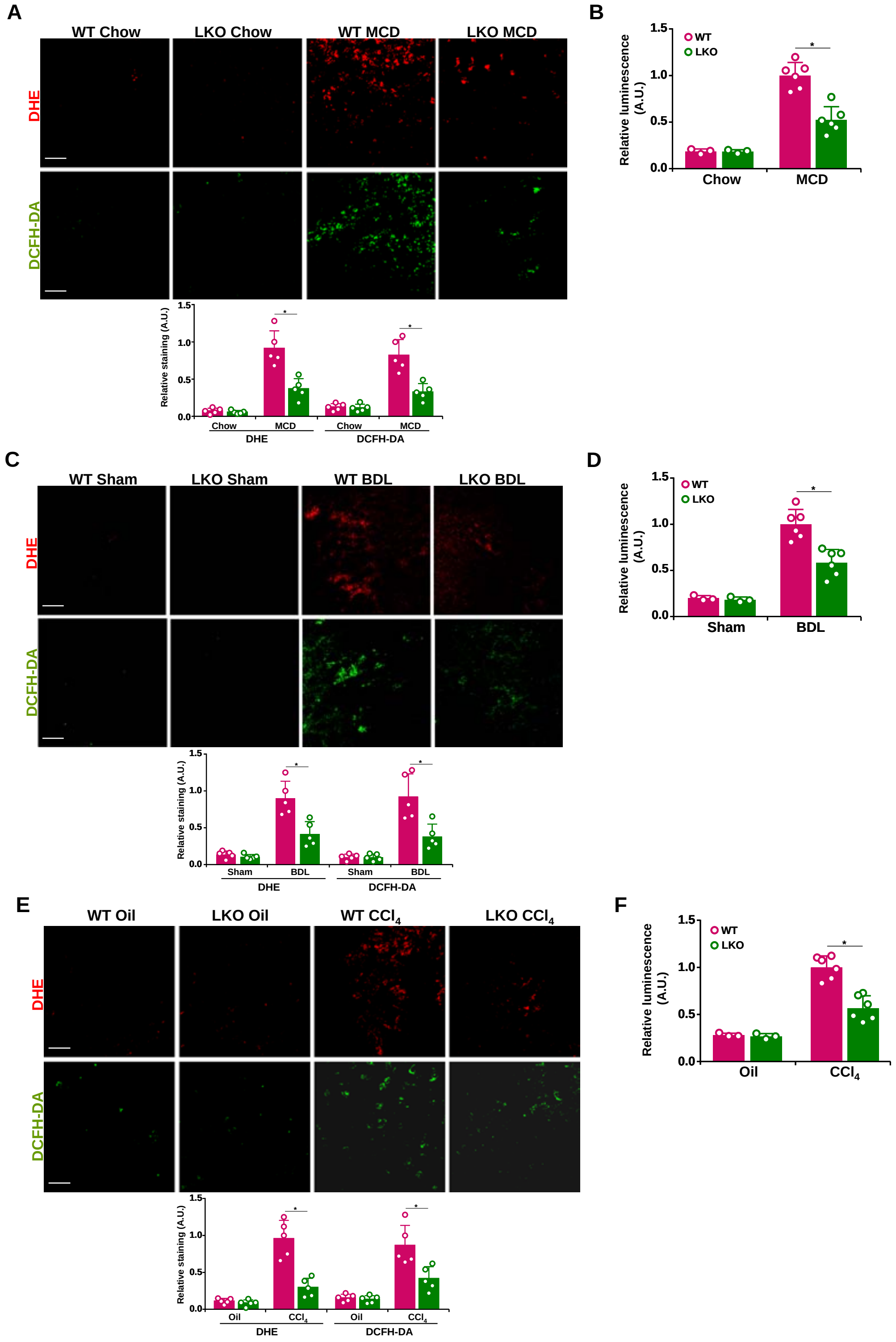

Figure 1: (**A, B**) BRG1 LKO and WT mice were subjected to the BDL procedure for 2wk as described in Methods. Frozen sections were stained with DHE or DFHC. ROS levels in the liver homogenates were measured with a fluorimetric kit. (**C, D**) BRG1 LKO and WT mice were fed an MCD diet for 4wk as described in Methods. Frozen sections were stained with DHE or DFHC. ROS levels in the liver homogenates were measured with a fluorimetric kit. (**E, F**) BRG1 LKO and WT mice were injected with $CCl_4$for 2wk as described in Methods. Frozen sections were stained with DHE or DFHC. ROS levels in the liver homogenates were measured with a fluorimetrickit. N=5 mice for each group.

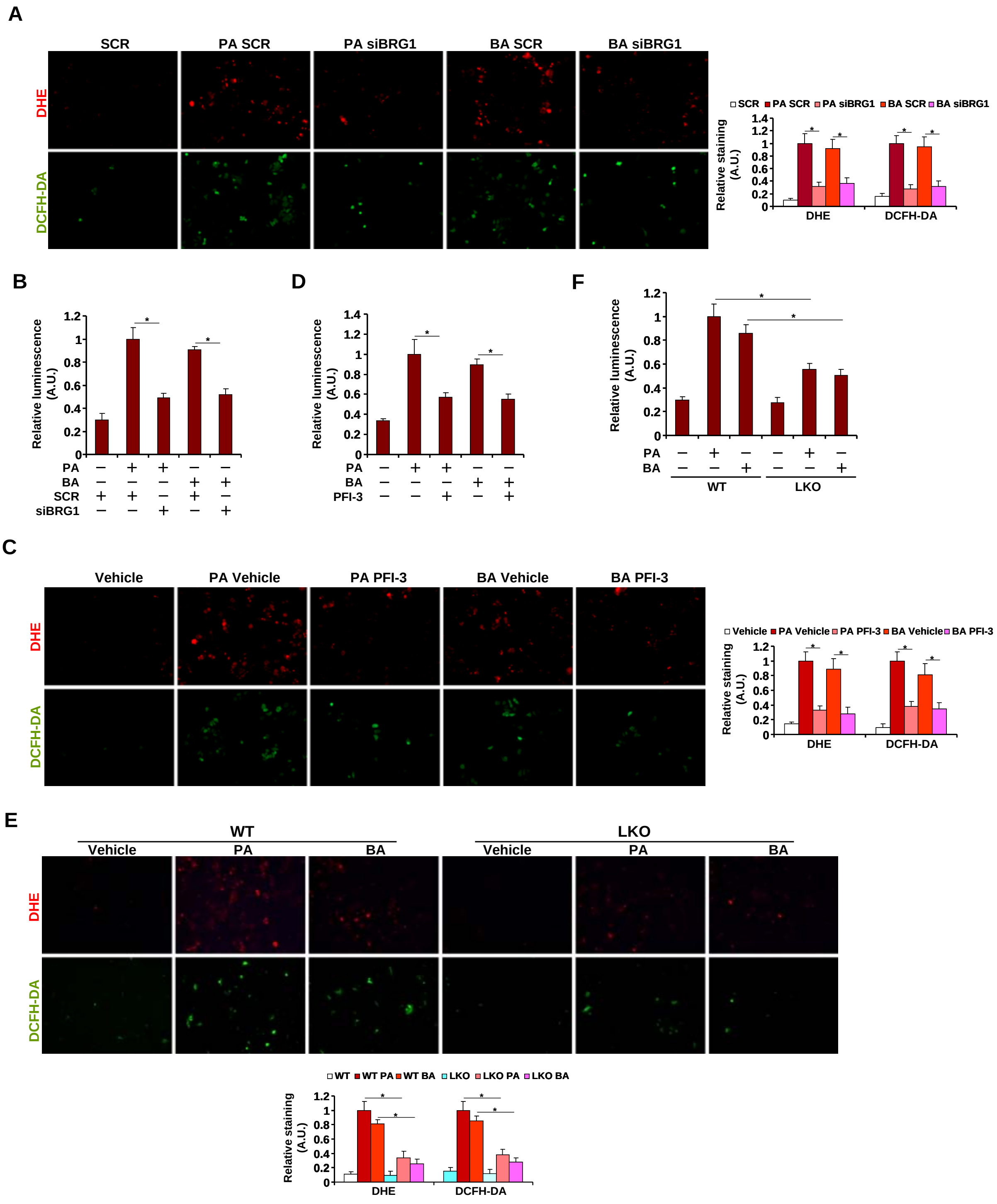

Figure S2: (**A, B**) HepG2 cells were transfected with siRNA targeting BRG1 or scrambled siRNA (SCR) followed by treatment with PA (0.4 mM) or BA (0.5 mM). ROS levels were examined by DHE staining, DCFH-DA staining, or a fluorimetric kit. (**C, D**) HepG2 cells were treated with PA (0.4 mM) or BA (0.5 mM)in the presence or absence of PFI-3 (5μM). ROS levels were examined by DHE staining, DCFH-DA staining, or a fluorimetric kit. (**E, F**) Primary hepatocytes were isolated from WT and LKO mice and exposed to PA (0.4 mM) or BA (0.5 mM). ROS levels were examined by DHE staining, DCFH-DA staining, or a fluorimetric kit.

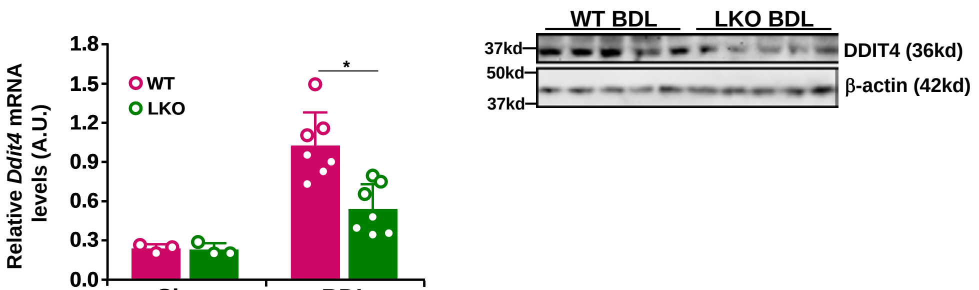

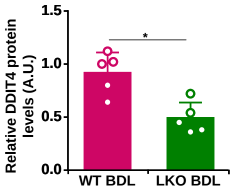

Fig.S3: BRG1 LKO and WT mice were subjected to the BDL procedure for 2wk as described in Methods. DDIT4 expression was examined by qPCR and Western.

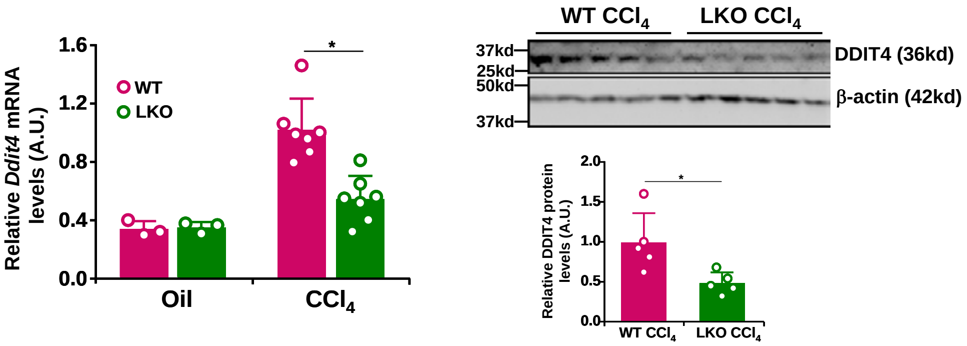

Fig.S4: BRG1 LKO and WT mice were injected with CCl_4 for 2wk as described in Methods. DDIT4 expression was examined by qPCR and Western.

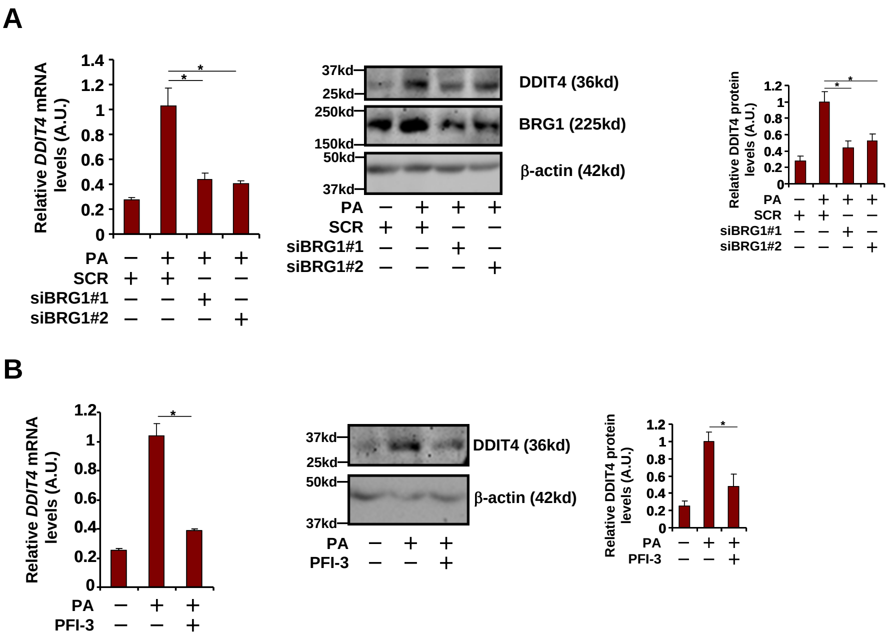

Fig.S5: (**A**) HepG2 cells were transfected with siRNA targeting BRG1 or scrambled siRNA (SCR) followed by treatment with PA (0.4 mM) for 24h. DDIT4 expression was examined by qPCR and Western. (**B**) HepG2 cells were treated with PA (0.4 mM) in the presence or absence of PFI-3 (5μM) for 24h. DDIT4 expression was examined by qPCR and Western.

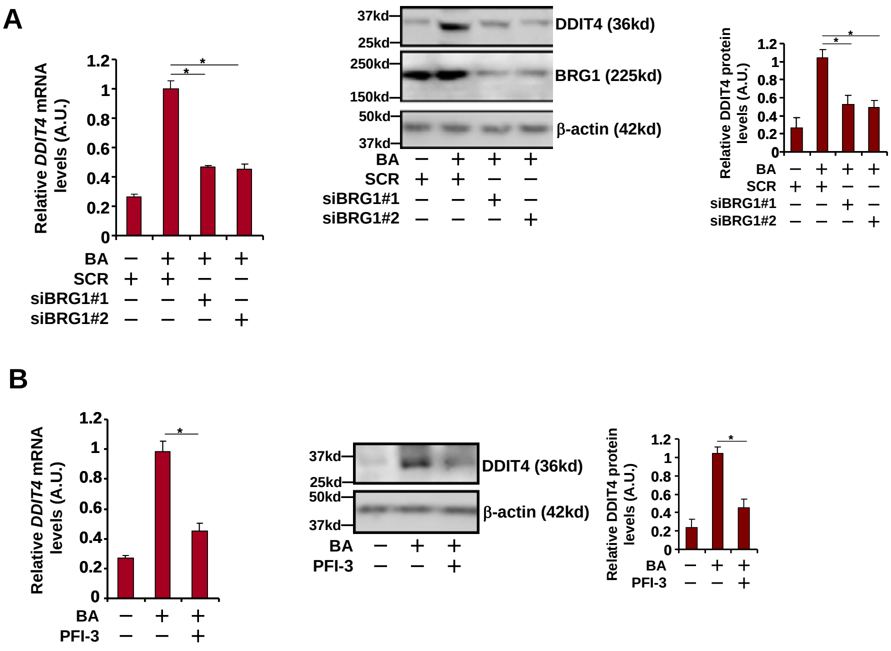

Fig.S6: (**A**) HepG2 cells were transfected with siRNA targeting BRG1 or scrambled siRNA (SCR) followed by treatment with BA (0.5 mM) for 24h. DDIT4 expression was examined by qPCR and Western. (**B**) HepG2 cells were treated with BA (0.5 mM) in the presence or absence of PFI-3 (5μM) for 24h. DDIT4 expression was examined by qPCR and Western.

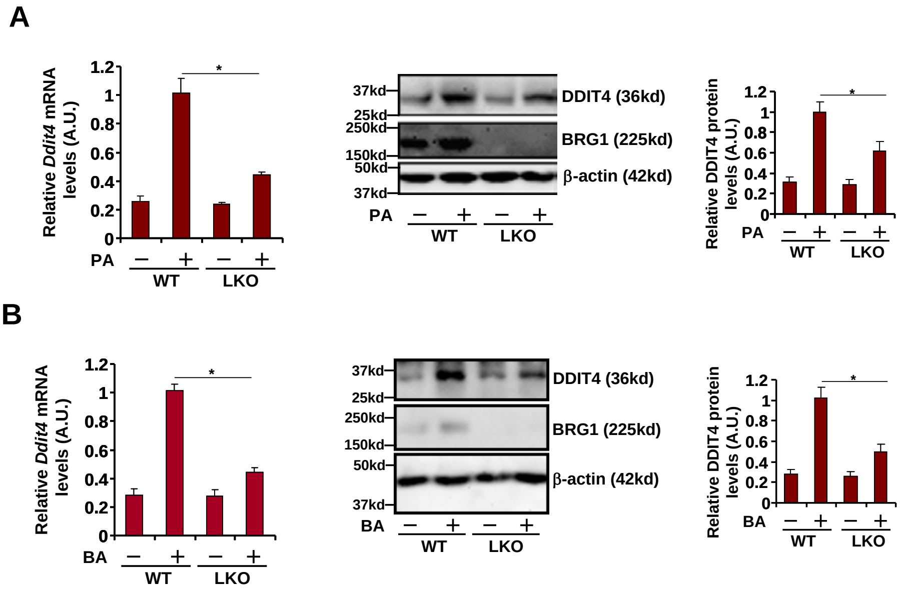

Fig.S7: (**A**) Primary hepatocytes were isolated from WT and LKO mice and exposed to PA (0.4 mM) for 24h. DDIT4 expression was examined by qPCR and Western. (**B**) Primary hepatocytes were isolated from WT and LKO mice and exposed to BA (0.5 mM) for 24h. DDIT4 expression was examined by qPCR and Western.

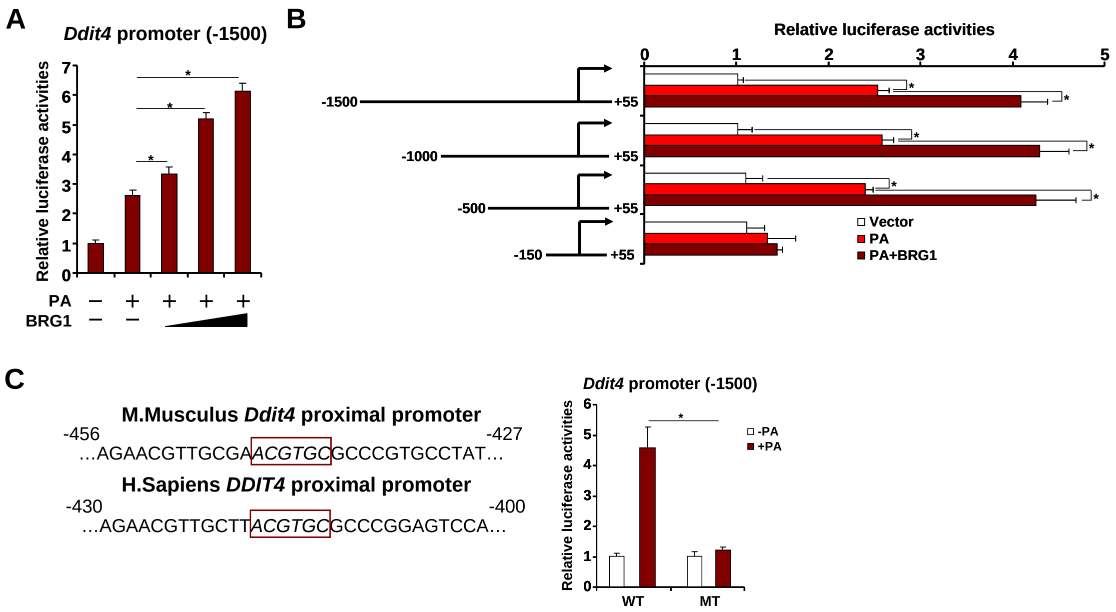

Fig.S8: (**A**) A DDIT4 promoter construct (-1500) was transfected into HepG2 cells with increasing doses of BRG1 followed by treatment with PA (0.4 mM). Luciferase activities were normalized by protein concentration and GFP fluorescence. (**B**) DDIT4 promoter constructs were transfected into HepG2 cells with or without BRG1 followed by PA (0.4 mM) treatment. Luciferase activities were normalized by protein concentration and GFP fluorescence. (**C**) Left panel: comparison of the human and the mouse DDIT4 proximal promoters. Right panel: wild type and mutant DDIT4 promoter constructs were transfected into HepG2 cells with or without BRG1 followed by PA (0.4 mM) treatment. Luciferase activities were normalized by protein concentration and GFP fluorescence.

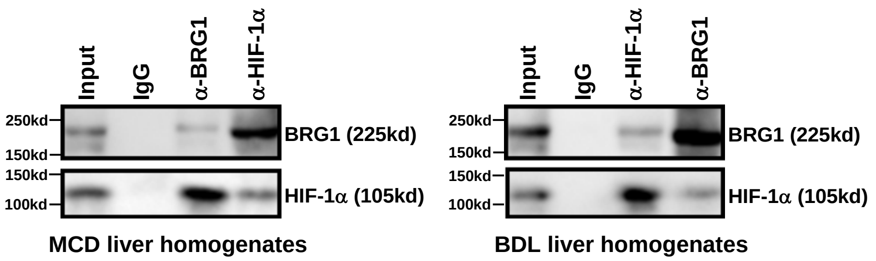

Fig.S9: Primary murine hepatocytes were exposed to PA (.4mM) or BA (.5mM) and harvested at indicated time points. ChIP assays were performed with anti-BRG1 or IgG.

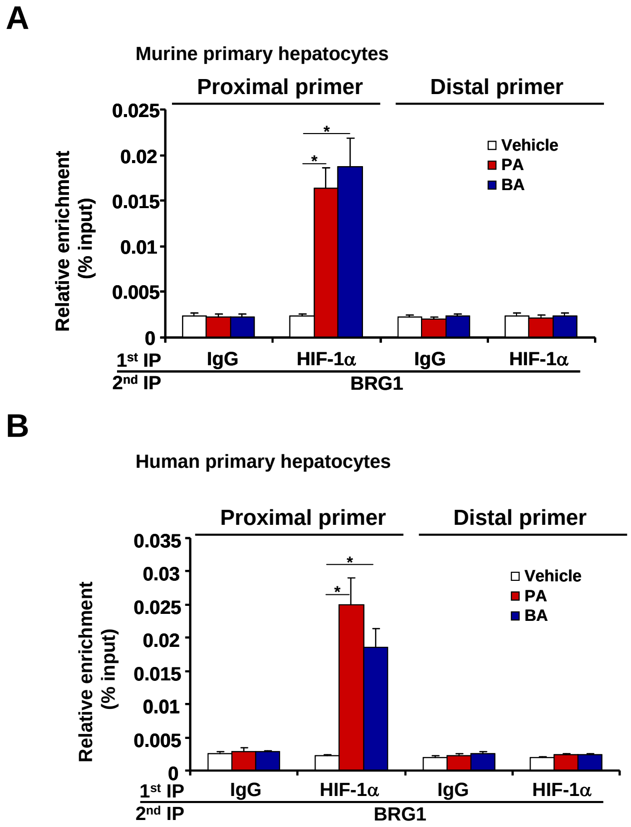

Fig.S10: (**A**) Primary murine hepatocytes were treated with PA (.4mM) or BA (.5mM) for 12h. Re-ChIP assays were performed with indicated antibodies. (**B**) Primary human hepatocytes were treated with PA (.4mM) or BA (.5mM) for 12h. Re-ChIP assays were performed with indicated antibodies.

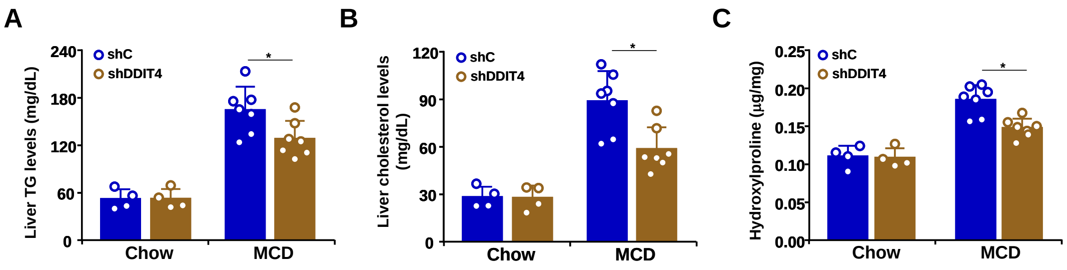

Fig.S11: C57/B6 mice were injected with adenovirus carrying DDIT4 shRNA or control adenovirus followed by MCD feeding for 4wk. (**A**) Hepatic triglyceride levels. (**B**) Hepatic cholesterol levels. (**C**) Hepatic hydroxylproline levels.

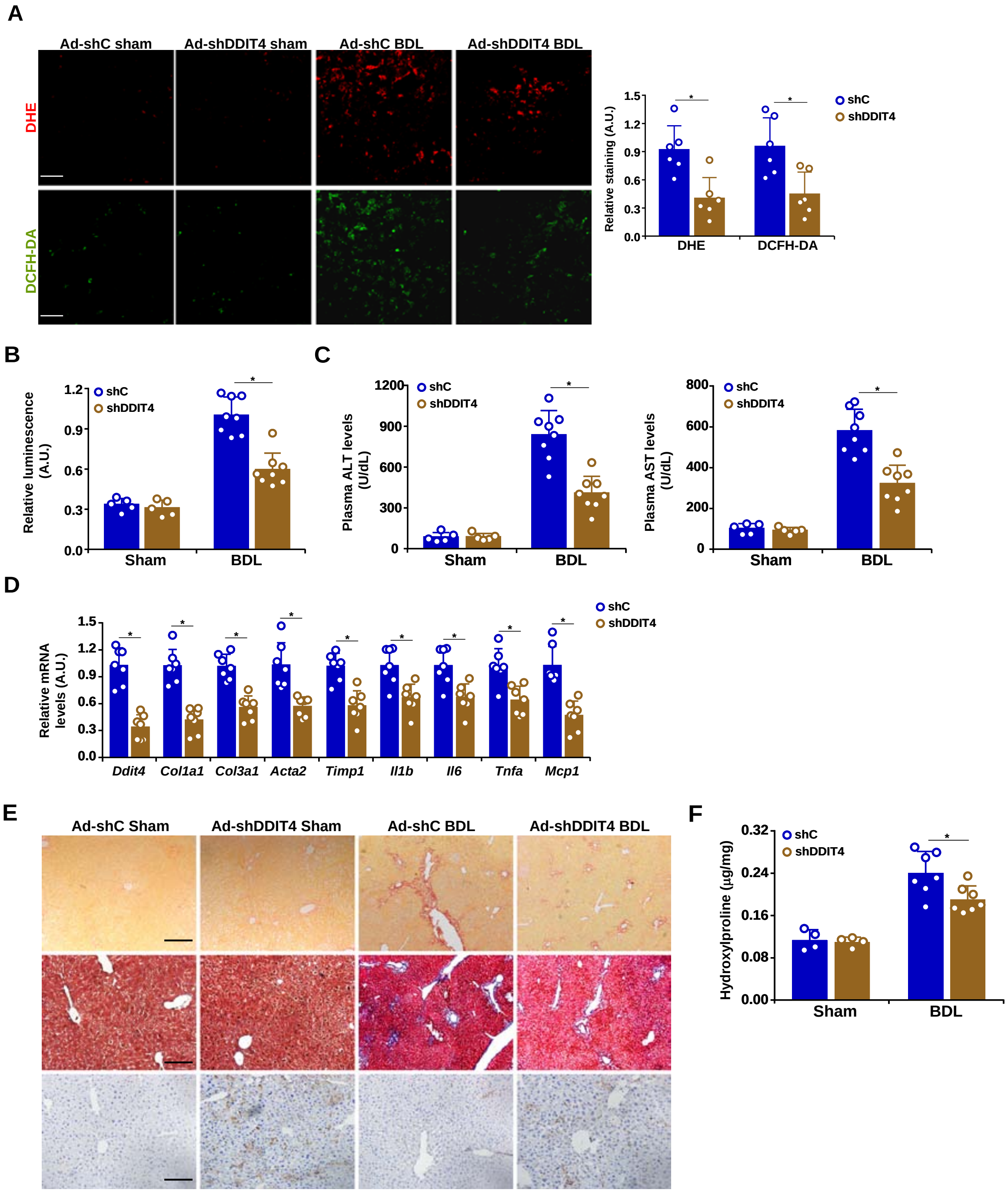

Fig.S12: C57/BL6 mice were injected via tail vein adenovirus carrying DDIT4 shRNA or control adenovirus followed by BDL for 2wk. (**A**) Frozen sections were stained with DHE or DFHC. (**B**) ROS levels in the liver homogenates were measured with a fluorimetric kit. (**C**) Plasma ALT and AST levels. (**D**) Gene expression levels were examined by qPCR. (**E**) Liver sections were stained with picrosirius red, Masson's trichrome, and anti-F4/80. (**F**) Hepatic hydroxylproline levels. N=5~8 mice for each group.

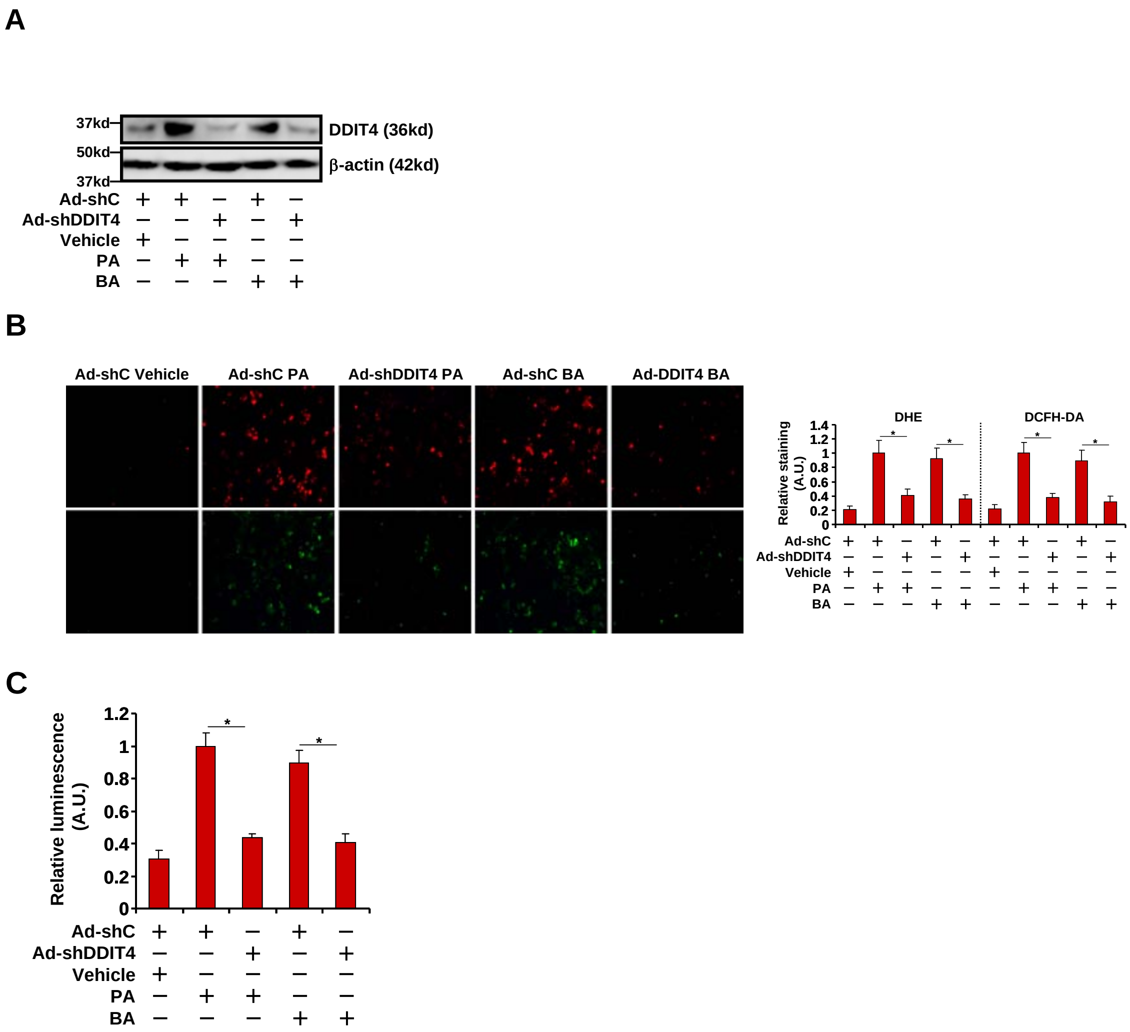

Fig.S13: Primary murine hepatocytes were infected with Ad-shC or Ad-shDDIT4 followed by treatment with PA or BA. (**A**) DDIT4 expression was examined by Western. (**B**) ROS levels were examined by DHE/DCFH-DA staining. (**C**) ROS levels were examined by fluorimetric kit.

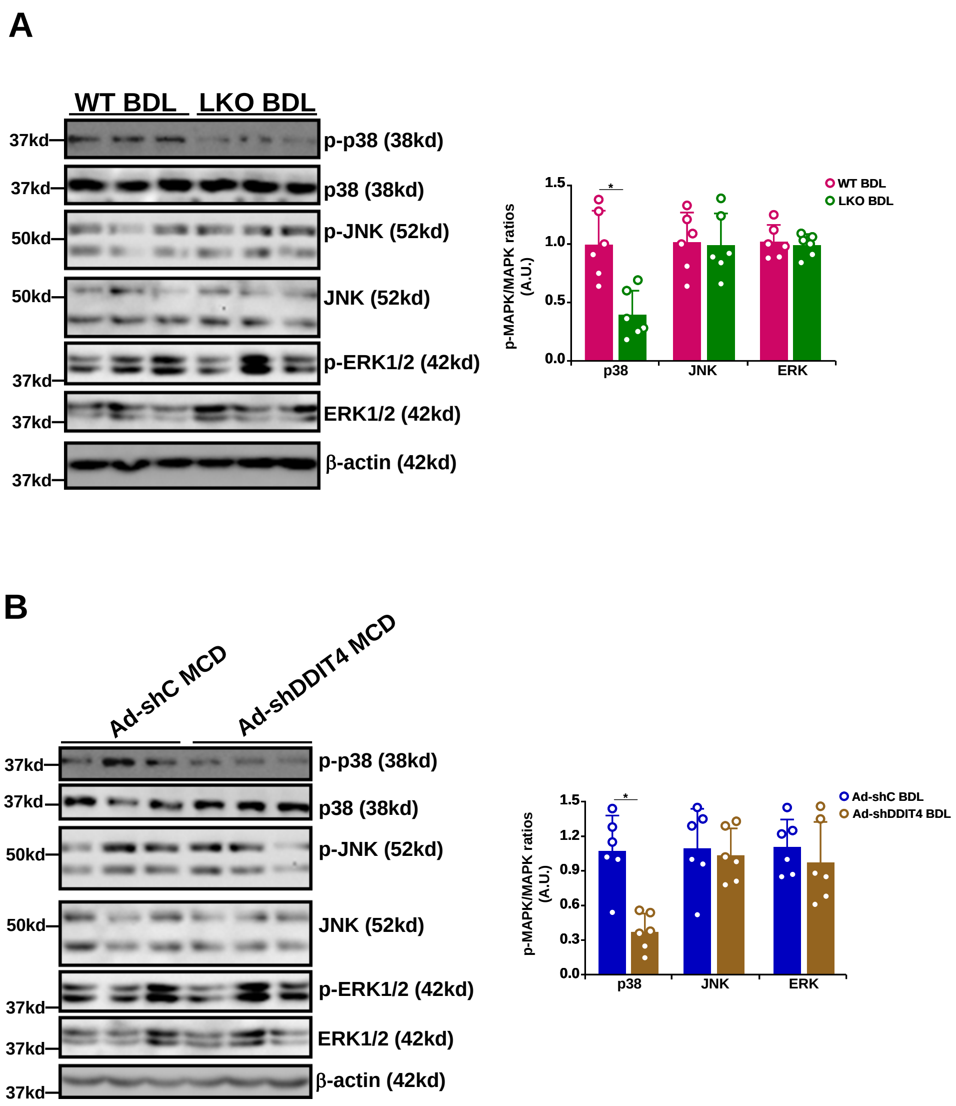

Fig.S14: (**A**) BRG1 LKO and WT mice were subjected to the BDL procedure for 2wk. MAPK phosphorylation was examined in liver lysates by Western. (**B**) C57/BL6 mice were injected via tail vein adenovirus carrying DDIT4 shRNA or control adenovirus followed by BDL for 2wk.MAPK phosphorylation was examined in liver lysates by Western.

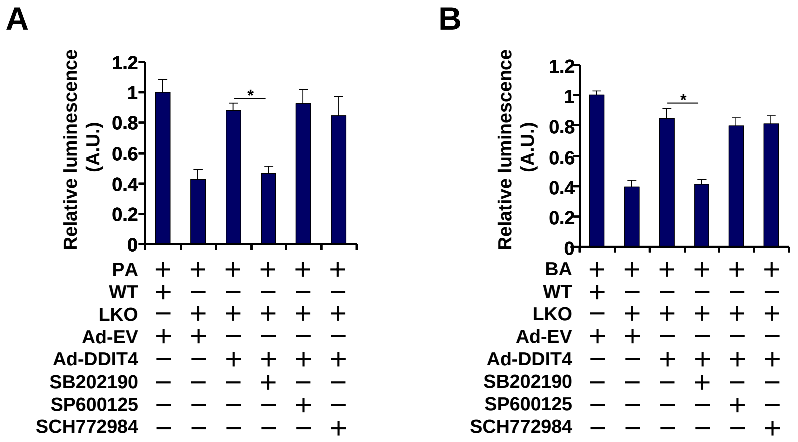

Fig.S15: (**A**) Primary hepatocytes were isolated from WT or BRG1 LKO mice and transduced with adenovirus carrying either DDIT4 expression vector (Ad-DDIT4) or an empty vector (Ad-EV) followed by treatment with PA (.4mM). ROS levels were examined a fluorimetric kit. (**A**) Primary hepatocytes were isolated from WT or BRG1 LKO mice and transduced with adenovirus carrying either DDIT4 expression vector (Ad-DDIT4) or an empty vector (Ad-EV) followed by treatment with BA (.5mM). ROS levels were examined a fluorimetric kit.

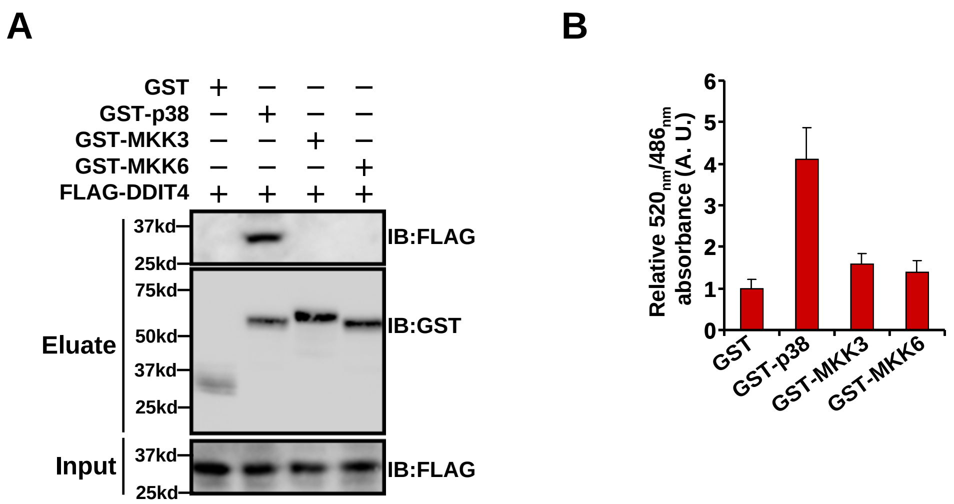

Fig.S16: (**A**) Primary murine hepatocytes were transduced with Ad-FLAG-DDIT4 followed by treatment with PA. Whole cell lysates were then incubated with purified GST proteins. GST pull-down assay was performed as described in Methods. (**B**) Primary murine hepatocytes were transduced with Ad-Venus-DDIT4 followed by treatment with PA. Whole cell lysates were then incubated with purified GST proteins and FRET assay was performed as described in Methods.

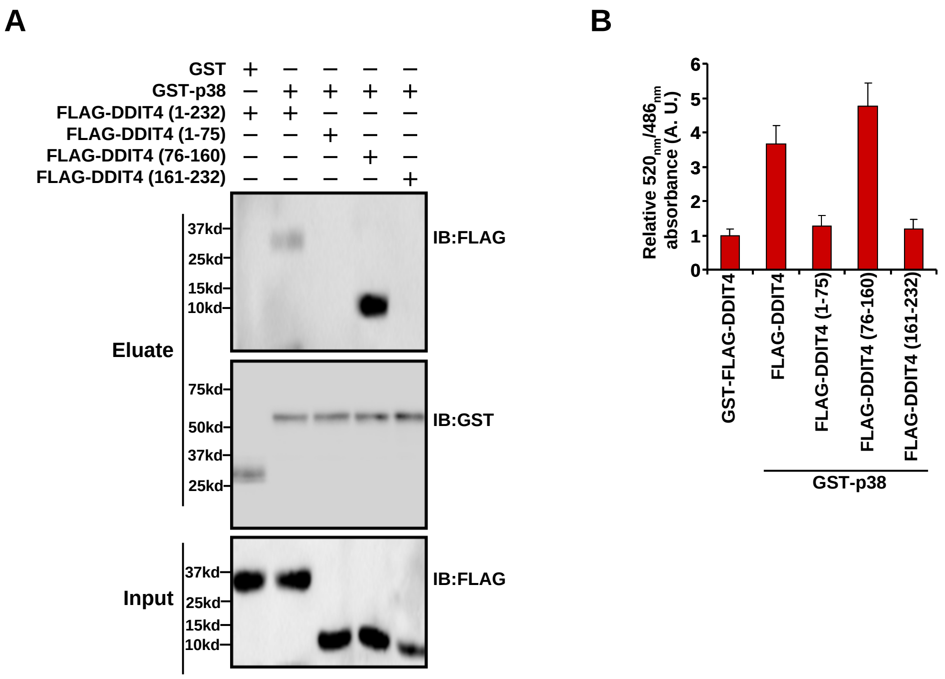

Fig.S17: (**A**) Primary murine hepatocytes were transduced with full-length or truncated Ad-FLAG-DDIT4 followed by treatment with PA. Whole cell lysates were then incubated with purified GST proteins. GST pull-down assay was performed as described in Methods. (**B**) Primary murine hepatocytes were transduced with full-length or truncated Ad-Venus-DDIT4 followed by treatment with PA. Whole cell lysates were then incubated with purified GST proteins and FRET assay was performed as described in Methods.

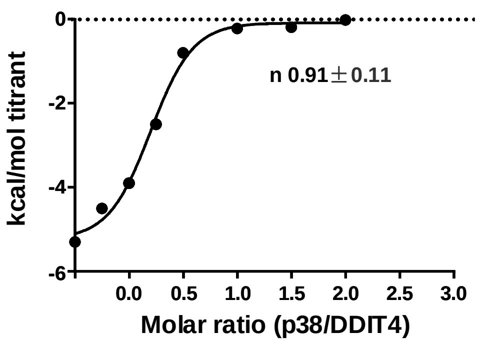

Fig.S18: Isothermal titration calorimetry was performed as described in the Methods. Data are fit using non-linear least squares.

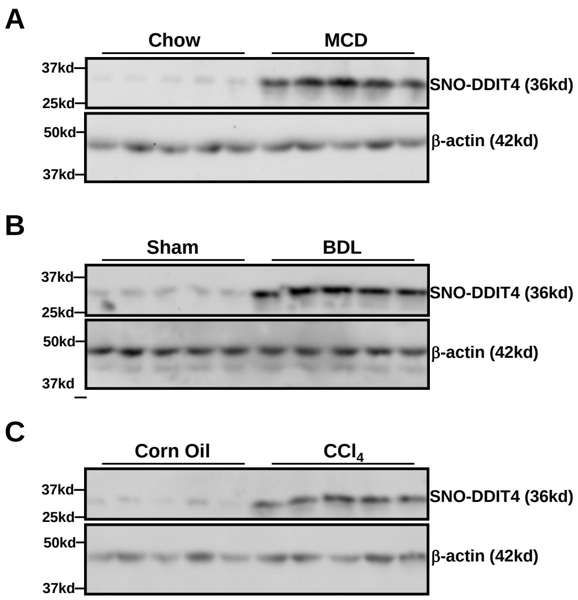

Fig.S19: (**A**) C57/BL6 mice were fed an MCD diet for 4wk as described in Methods. DDIT4 S-nitrosylation was determined by biotin exchange assay followed by Western blotting. (**B**) C57/BL6 mice were subjected to the BDL procedure for 2wk as described in Methods. DDIT4 S-nitrosylation was determined by biotin exchange assay followed by Western blotting. (**C**) C57/BL6 mice were injected with $CCl_4$for 2wk as described in Methods. DDIT4 S-nitrosylation was determined by biotin exchange assay followed by Western blotting. N=5 mice for each group.

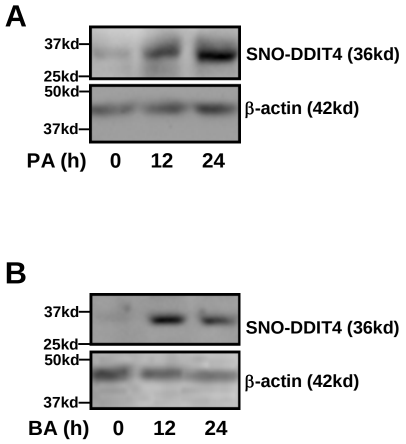

Fig.S20: (**A**) Primary murine hepatocytes were exposed to PA (.4mM) and harvested at indicated time points. DDIT4 S-nitrosylation was determined by biotin exchange assay followed by Western blotting. (**B**) Primary murine hepatocytes were exposed to BA (.5mM) and harvested at indicated time points. DDIT4 S-nitrosylation was determined by biotin exchange assay followed by Western blotting.

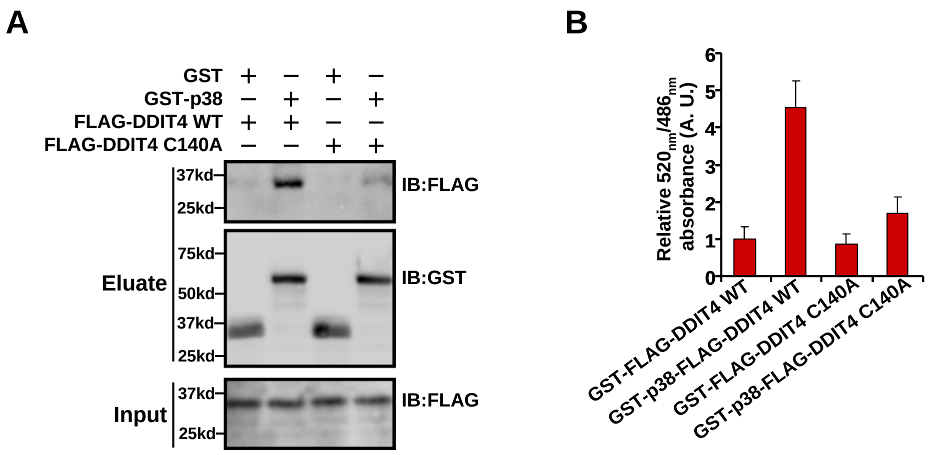

Fig.S21: (**A**) Primary murine hepatocytes were transduced with wild type or mutant Ad-FLAG-DDIT4 followed by treatment with PA. Whole cell lysates were then incubated with purified GST proteins. GST pull-down assay was performed as described in Methods. (**B**) Primary murine hepatocytes were transduced with wild type or mutant Ad-Venus-DDIT4 followed by treatment with PA. Whole cell lysates were then incubated with purified GST proteins and FRET assay was performed as described in Methods.

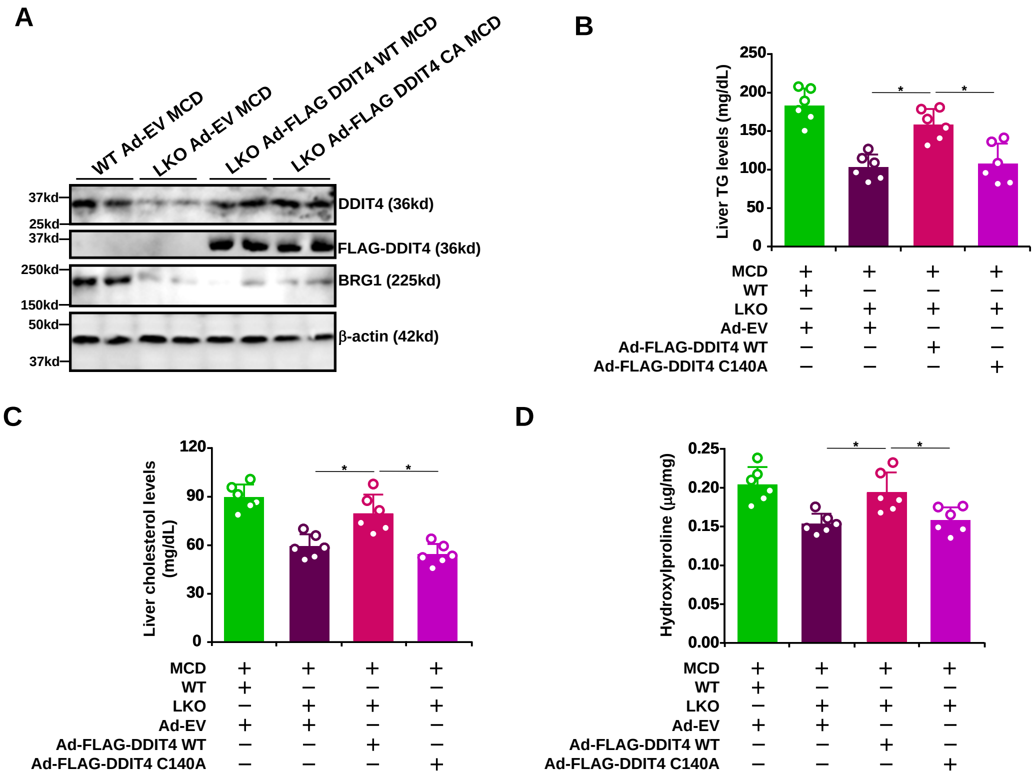

Fig.S22: BRG1 LKO mice were injected via tail vein Ad-FLAG-DDIT4 or Ad-EV followed by MCD feeding along with the WT mice for 4wk. (**A**) DDIT4 expression in the livers was examined by Western. (**B**) Hepatic triglyceride levels. (**C**) Hepatic cholesterol levels. (**D**) Hepatic hydroxylproline levels.

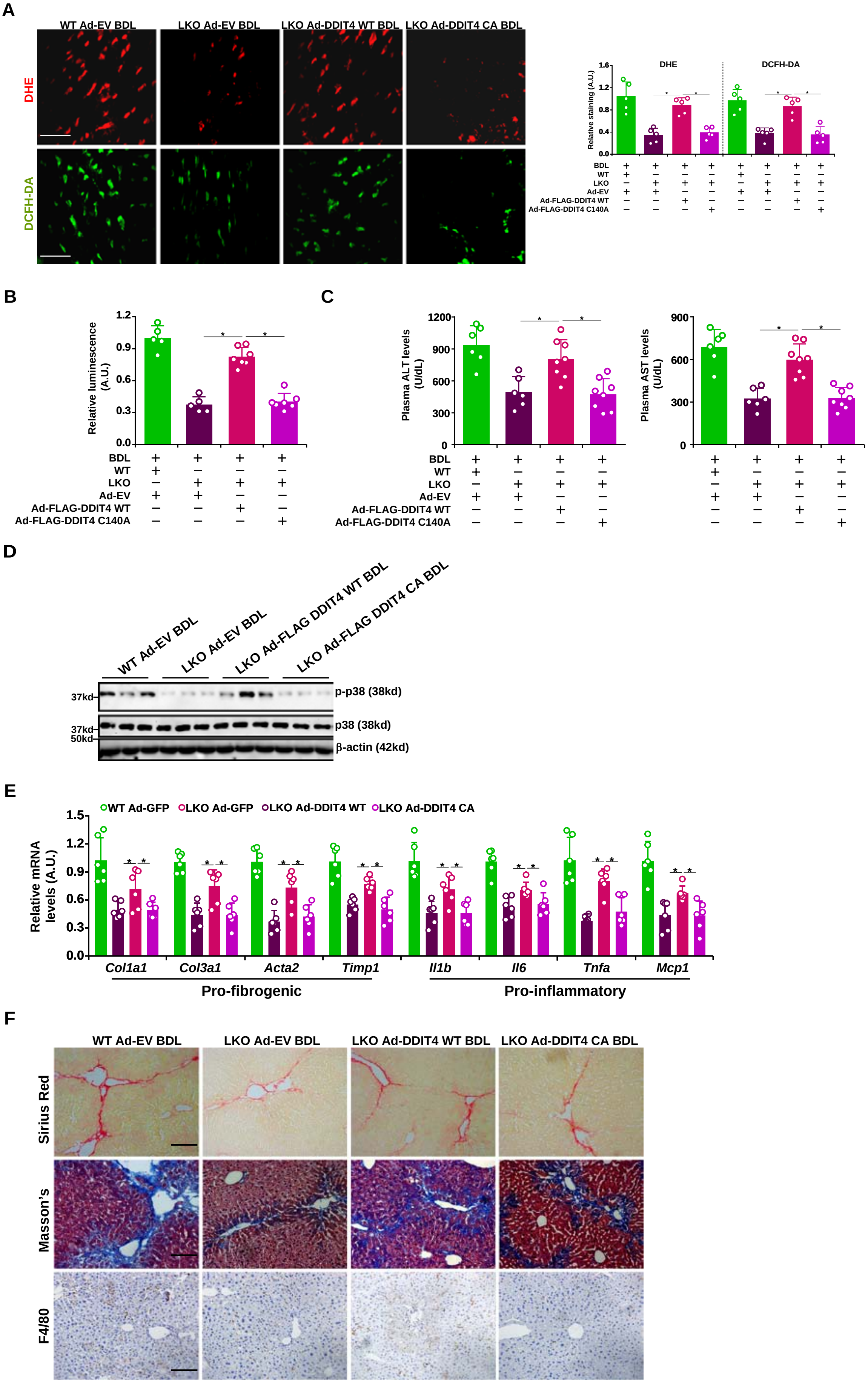
A
WT Ad-EV BDL
LKO Ad-EV BDL
LKO Ad-DDIT4 WT BDL
LKO Ad-DDIT4 CA BDL
DHE
DCFH-DA
Relative staining (A.U.)
BDL
WT
LKO
Ad-EV
Ad-FLAG-DDIT4 WT
Ad-FLAG-DDIT4 C140A
B
Relative luminescence (A.U.)
C
Plasma ALT levels (U/dL)
Plasma AST levels (U/dL)
D
WT Ad-EV BDL
LKO Ad-EV BDL
LKO Ad-FLAG DDIT4 WT BDL
LKO Ad-FLAG DDIT4 CA BDL
37kd
50kd
p-p38 (38kd)
p38 (38kd)
β-actin (42kd)
E
WT Ad-GFP
LKO Ad-GFP
LKO Ad-DDIT4 WT
LKO Ad-DDIT4 CA
Relative mRNA levels (A.U.)
Col1a1
Col3a1
Acta2
Timp1
Il1b
Il6
Tnfa
Mcp1
Pro-fibrogenic
Pro-inflammatory
F
WT Ad-EV BDL
LKO Ad-EV BDL
LKO Ad-DDIT4 WT BDL
LKO Ad-DDIT4 CA BDL
Sirius Red
Masson's
F4/80

G

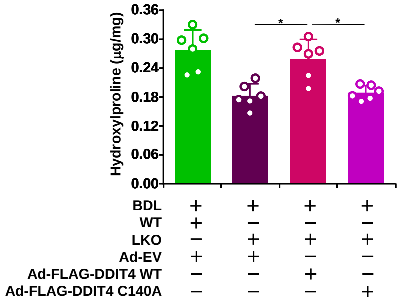

Fig.S23: BRG1 LKO mice were injected via tail vein Ad-FLAG-DDIT4 or Ad-EV followed by the BDL procedure along with the WT mice for 2wk. (**A**) Frozen sections were stained with DHE or DFHC. (**B**) ROS levels in the liver homogenates were measured with a fluorimetric kit. (**C**) Plasma ALT and AST levels. (**D**) MAPK phosphorylation was examined by Western. (**E**) Gene expression levels were examined by qPCR. (**F**) Liver sections were stained with picrosirius red, Masson's trichrome, anti-F4/80, and oil red O. (**G**) Hepatic hydroxylproline levels. N=6-8 mice for each group.

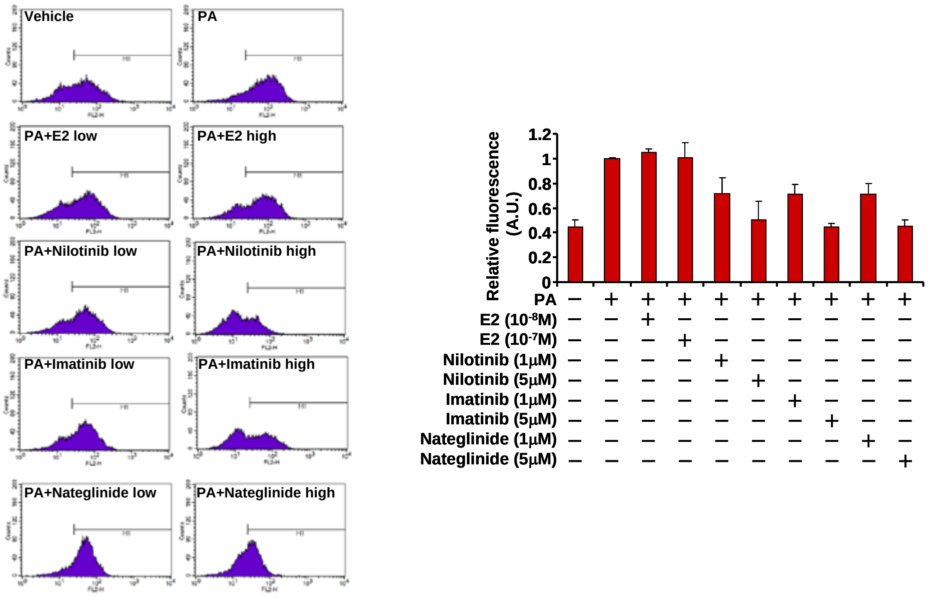

Fig.24: Primary murine hepatocytes were treated with PA (0.4 mM) in the presence or absence of indicated compounds. ROS levels were examined by DHE labeling and flow cytometry.

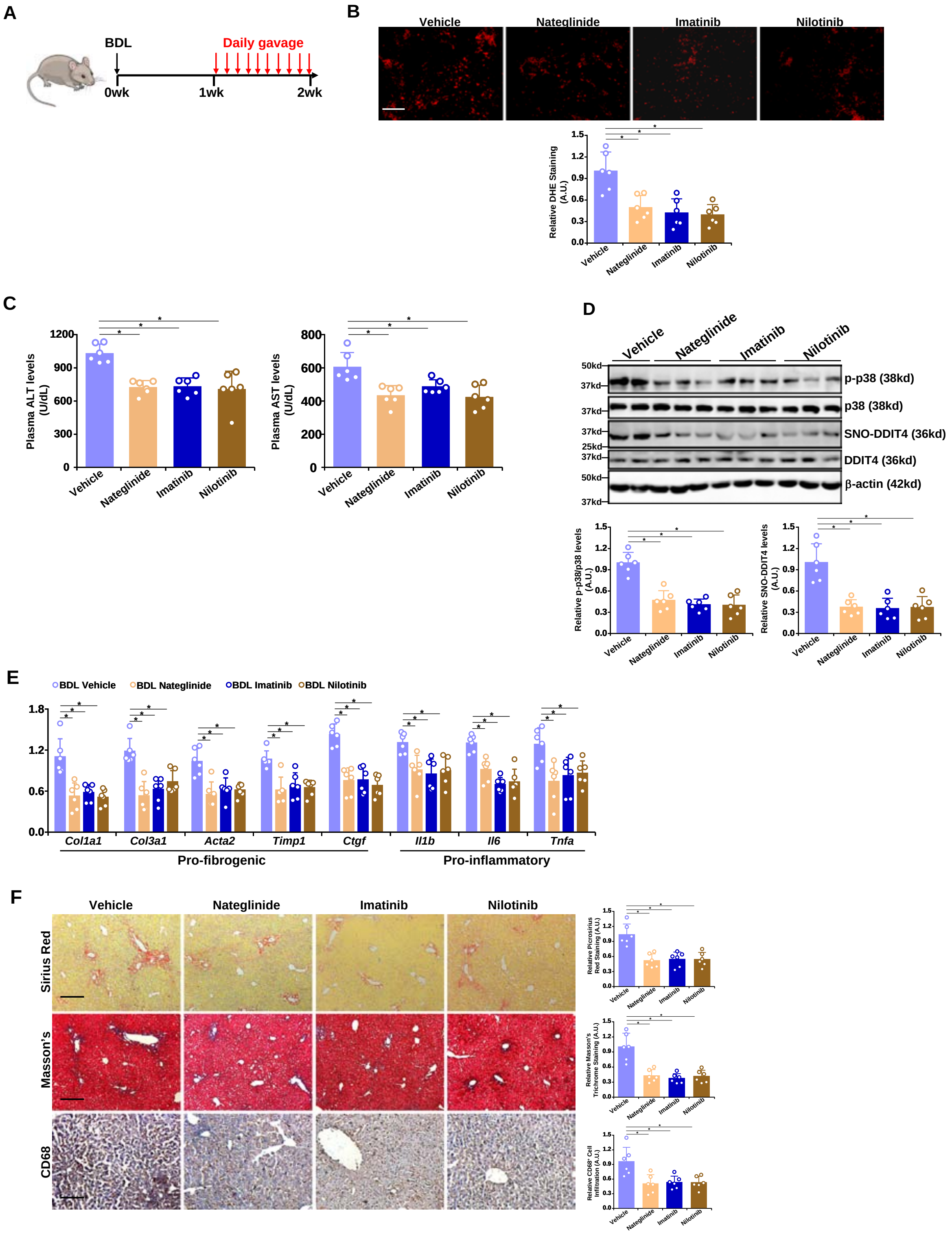

Fig.S25: (**A**) Schematic protocol. (**B**) Frozen sections were stained with DHE. (**C**) Plasma ALT and AST levels. (**D**) MAPK phosphorylation was examined by Western. (**E**) Gene expression levels were examined by qPCR. (**F**) Liver sections were stained with picrosirius red, Masson's trichrome, and anti-CD68. N=6 mice for each group.

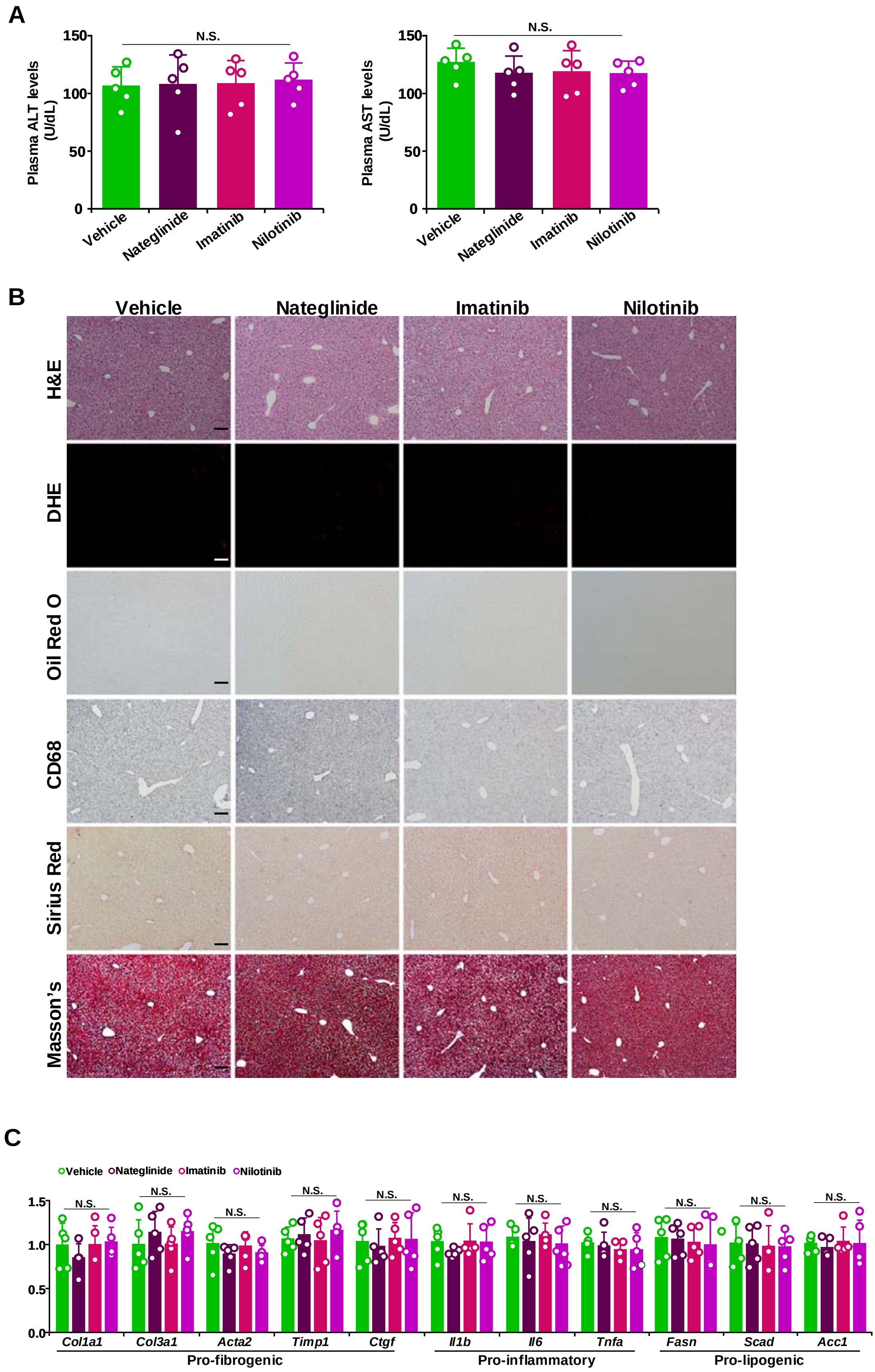

A
Plasma ALT levels (U/dL)
Plasma AST levels (U/dL)
N.S.
150
100
50
0
Vehicle
Nateglinide
Imatinib
Nilotinib
B
Vehicle
Nateglinide
Imatinib
Nilotinib
H&E
DHE
Oil Red O
CD68
Sirius Red
Masson's
C
Vehicle
Nateglinide
Imatinib
Nilotinib
1.5
1.0
0.5
0.0
Col1a1
Col3a1
Acta2
Timp1
Ctgf
Il1b
Il6
Tnfa
Fasn
Scad
Acc1
Pro-fibrogenic
Pro-inflammatory
Pro-lipogenic

D

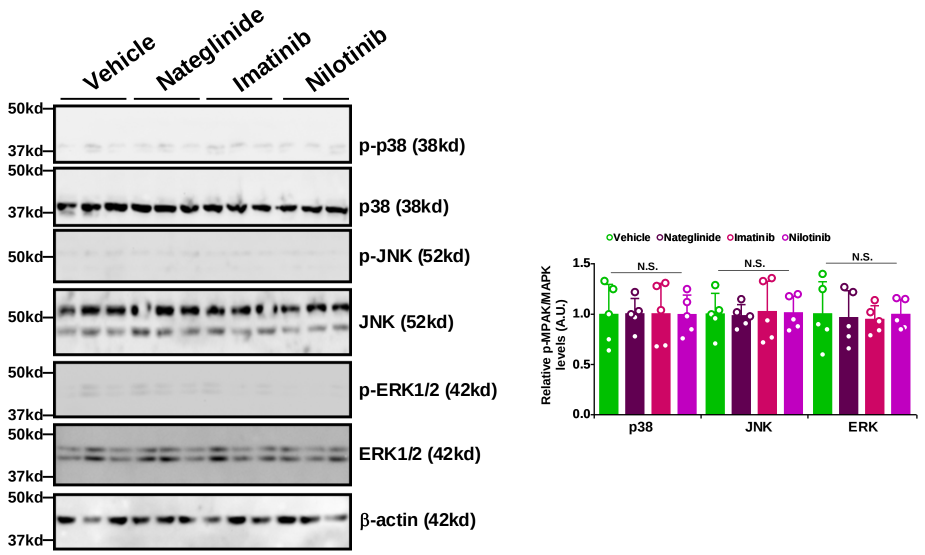

Fig.S26: 6~8 week-old male C57B6/L mice were given Imatinib, Nilotinib, and Nateglinide through daily gavage for three consecutive weeks. (**A**) Plasma ALT and AST levels. (**B**) MAPK phosphorylation was examined by Western. (**E**) Gene expression levels were examined by qPCR. (**F**) Liver sections were stained with picrosirius red, Masson's trichrome, and anti-CD68. N=5 mice for each group.

Table I: qPCR primers sequences

Gene	Forward primer	Reverse primer
Mouse Ddit4	CCTGCGCGTTTGCTCATGCC	GGCCGCACGGCTCACTGTAT
Human DDIT4	TGAGGATGAACACTTGTGTGC	CCAACTGGCTAGGCATCAGC
Human BRG1	GAGGAGGTCCGGCAGAAGAAATC	TTCTTCTGCTTCTTGCTCTC
Mouse Ddit4 promoter proximal	ACCAGGCAGGAGAGAACGTT	AGCCCAATCGAGACCCGGGAG
Mouse Ddit4 promoter distal	AATTGAATTCTCCTGCCAC	AAACCCACTGCGCAGAGC

Table II: antibody information

Antigen	Vendor (catalog#)	Application
DDIT4	Proteintech (10638-1-AP)	Western, IP
BRG1	Santa Cruz (sc-17796)	Western, IP, ChIP
HIF-1α	Santa Cruz (sc-10790)	Western, IP, ChIP
p38	Abcam (ab170099)	Western, IP
p-p38	Cell Signaling Tech (4511)	Western
JNK	Proteintech (24164-1-AP)	Western
p-JNK	Cell Signaling Tech (9251)	Western
ERK1/2	Cell Signaling Tech (5013)	Western
p-ERK/12	Cell Signaling Tech (9101)	Western
MKK3	Abcam (ab195037)	Western
MKK6	Abcam (ab33866)	Western
β-actin	Sigma (A2228)	Western
